# Optimal Sensor Placement for Reliable Virtual Sensing Using Modal Expansion and Information Theory

**DOI:** 10.3390/s21103400

**Published:** 2021-05-13

**Authors:** Tulay Ercan, Costas Papadimitriou

**Affiliations:** Department of Mechanical Engineering, University of Thessaly, Pedion Areos, 383 34 Volos, Greece; ercan@uth.gr

**Keywords:** information gain, Kullback-Leibler divergence, relative entropy, Bayesian inference, response predictions

## Abstract

A framework for optimal sensor placement (OSP) for virtual sensing using the modal expansion technique and taking into account uncertainties is presented based on information and utility theory. The framework is developed to handle virtual sensing under output-only vibration measurements. The OSP maximizes a utility function that quantifies the expected information gained from the data for reducing the uncertainty of quantities of interest (QoI) predicted at the virtual sensing locations. The utility function is extended to make the OSP design robust to uncertainties in structural model and modeling error parameters, resulting in a multidimensional integral of the expected information gain over all possible values of the uncertain parameters and weighted by their assigned probability distributions. Approximate methods are used to compute the multidimensional integral and solve the optimization problem that arises. The Gaussian nature of the response QoI is exploited to derive useful and informative analytical expressions for the utility function. A thorough study of the effect of model, prediction and measurement errors and their uncertainties, as well as the prior uncertainties in the modal coordinates on the selection of the optimal sensor configuration is presented, highlighting the importance of accounting for robustness to errors and other uncertainties.

## 1. Introduction

Virtual sensing is used to complement the physical sensing when the direct observations are not available in field and laboratory experiments. Virtual sensing is accomplished by combining the information in output-only vibration measurements with the information contained in a model (usually a finite element model) of the system to predict response time histories of various quantities of interest (QoI). The subject of virtual sensing, also known as response reconstruction, has received a lot of attention recently due to its importance in monitoring critical performance and safety-related quantities such as accelerations, displacements, structural shapes, interstory drifts, strains/stresses and fatigue damage accumulation in structures that operate in a dynamic environment.

Filtering techniques for input, state and parameter estimation as well as modal expansion techniques for response reconstruction are the two type of methods extensively used in the literature for virtual sensing and response reconstruction. The optimal sensor placement techniques developed in this work are based on modal expansion techniques for virtual sensing. The modal expansion technique represents the system response QoI (acceleration, displacement, strain, etc.) as a modal superposition involving the modeshapes of the structure (e.g., displacement or strain modeshapes) and a fixed number of modal coordinates. This allows the prediction (virtual sensing) of any response QoI by estimating the temporal variability of the modal coordinates from measured response time histories and borrowing the information from a finite element model for representing the rest of the quantities involved in the modal expansion.

The modal expansion method has been used in structural dynamics for reconstructing stress/strain fields using limited number of acceleration measurements [1] or displacement/strain measurements [2,3,4]. It is pointed out that estimating the strains/stresses is important for fatigue damage identification. The potential of providing fatigue damage accumulation predictions in the entire body of metallic structures based on virtual strain/stress sensing has been demonstrated for the first time in [5,6] by combining output-only vibration measurements, finite element models and filtering techniques with stochastic and deterministic fatigue theories. Such predictions are based on the actual operating conditions of structures and thus provide realistic fatigue estimates consistent with existing fatigue theories.

Following these works, the modal expansion and filtering techniques were applied for strain/stress virtual sensing [3,7,8,9,10,11,12,13,14,15] and fatigue estimation [16,17,18,19,20,21] for a number of structures using limited number of displacement/strain physical sensors. In particular, modal expansion techniques have been used in mechanical and aerospace systems for shape and/or strain reconstruction using sparse displacement and/or strain measurements [22,23,24,25,26,27,28,29] or fusing acceleration and strain measurements [30]. The aforementioned studies cover a number of applications, including components of mechanical structures [1,2], components of civil structures such as railway bridges [20], wind turbine jacket substructures [11], truss structures [12], wind turbine towers [7,8,9,10,16,25], wind turbine blades [27,28], offshore structures [11], components of industrial structures [17,21], roller coaster [18], rotating [26] and underwater structures [13], as well as biologicaly inspired wing structures for robotic applications [29]. Recently, it is suggested to use virtual sensing in isolated linear components of linear and nonlinear models of structures [31,32,33,34] considering the forces at the interface between the analyzed linear component and the rest of the structure as unknown forces. In a limited number of past studies the effect of the number and location of sensors, as well as the measurement error, on the accuracy of the response reconstruction was investigated and its importance was pointed out [2,22,23,24,35].

Bayesian methods were applied for modal identification and virtual sensing using modal expansion techniques to better account for the model and measurement errors in the modal coordinates estimation [36] and response predictions [37,38,39] and also used for damage detection [40,41,42]. Such methods have the advantage of predicting also the uncertainty in the modal coordinates and/or predictions. In particular, the uncertainty formulation is useful for optimizing the location and number of sensors by minimizing the uncertainty in the estimates of the modal coordinates [36] and response predictions or virtual sensing [38,43,44].

OSP techniques have been developed in the past for the purpose of extracting the most informative data from a given number of sensors. A recent article [45] reviews methods and optimization algorithms for optimizing the location of sensor in a structure. Selecting the most informative sensor configuration is often performed using information theory based approaches. Measures of information that have been used in the past for structural dynamics problems include the Fisher information matrix (FIM) [46,47,48,49,50,51], the information entropy [52,53,54,55,56,57,58,59,60,61,62,63,64], the joint entropy [65,66], the expected Kullback–Liebler divergence (KL-div) index [67,68,69] and mutual information [70,71], and the value of information [72,73,74]. FIM-based OSP techniques address problems of parameter estimation of physics-based models for linear structural systems [46,47,48], modal estimation [51], as well as identification of distributed parameter systems [49,50]. The OSP techniques based on information and joint entropy address problems of parameter estimation of linear systems subjected to known [54,55,56,63,64] and unknown [53] excitation, model updating [52,61], modal identification [59], model selection [58], structural health monitoring and damage detection [60,62]. In particular, techniques to avoid sensor clustering by taking into account the redundant information contained in the measurements have been discussed in [57,59]. OSP approaches based on expected KL-div and utility theory also address problems of parameter estimation of linear [70,75,76,77] and nonlinear [78] structural systems, modal estimation [71,79] and structural health monitoring [80,81]. Finally, OSP issues related to multi-type sensor placement have also been addressed [56,65]. The aforementioned methods, however, have focused on OSP problems for parameter estimation of physics-based or mathematical models. Using information theory formulations, the OSP problem for response reconstruction has been studied recently considering that the input forces are known [38,44]. OSP for response reconstruction under unknown input forces modeled by stochastic processes has been attempted in [82] using the kriging technique. An OSP formulation to handle the case of output-only vibration measurements has also been presented recently by combining filtering techniques with information theory [43].

In this work, considering output-only vibration measurements, a novel OSP framework is presented for accurate response reconstruction and virtual sensing in linear systems based on modal expansion techniques and information theory. The information gained by a sensor configuration is measured by the KL-div [83] between the posterior and prior probability distribution of the response QoI to be virtually sensed or reconstructed, by combining available modal expansion techniques and data. The KL-div is averaged over all possible QoI to be sensed. This is obtained using the Lindley’s utility function [68,84] quantifying the average information in the data over all possible measurements generated by the prediction error model. The measure is extended to include uncertainties in the model parameters, as well as in the model/prediction and measurement errors which are assigned in the modeling process for the Bayesian estimation of the posterior distribution of the modal coordinates. The optimal sensor configuration is obtained by maximizing the utility function. For the case of uncertainties in model parameters, the utility function involves a multidimensional integral over the uncertain parameter space which can be computed using sparse grid or Monte Carlo techniques. Due to the linearity of the response QoI and the modal coordinates, exact analytical expressions are developed for the utility function in terms of the variance of the responses of the QoI to be sensed. The structure of the analytical expressions developed are used to derive useful formulas that show the effect of measurements and model/prediction errors on the expected information gained from the data, as well as derive the dependence of the information gain as a function of the number of sensors.

This study is organized as follows: in Section 2, the modal expansion is outlined for formulating the uncertainty in the predictions of response QoI. In Section 3, the optimal sensor placement methodology for predicting response time histories of desirable QoI (virtual sensing) with the least uncertainty is presented based on utility and information theory. In Section 4, models for the model/prediction and measurement errors required in the formulation are introduced. Section 5 discusses implementation issues and the importance of taking into account the uncertainties in the input characteristics for optimizing the sensor placement. An application on a square plate structure is used in Section 6 to demonstrate the capabilities and effectiveness of the OSP methodology for reliable virtual sensing. Conclusions are drawn in Section 7.

## 2. Bayesian Virtual Sensing Using the Modal Expansion Method

Consider a structural model used to predict the temporal variability of the response vector $\underline{z}\left(t;\underline{\varphi }\right)\in R^{n_{z}}$ (e.g., accelerations, displacements, strain or stresses) at $n_{z}$ locations given the values of a structural model parameter set $\underline{\varphi }$ (e.g., stiffness, mass and damping related parameters) and the excitation vector $\underline{u}\left(t\right)\in R^{n_{u}}$. Let $D=\left\{\underline{y}\left(t\right)\in R^{N_{0}}\right\}$ be the vector of response time history data collected by placing $N_{0}$ sensors in the structure. These data depend on the sensor configuration vector $\underline{\delta }\in R^{N_{0}}$ indicating the location and measurement direction of sensors placed in a structure. The data may consist of either acceleration, displacement and strain measurements. In what follows, a linear model of the structure is assumed. Additionally, it is assumed that the excitation time histories $\underline{u}\left(t\right)$ are not available.

### 2.1. Modal Expansion for Virtual Sensing

Given output-only data $\underline{y}\left(t\right)$, the modal expansion technique is used to predict responses at measured and unmeasured output QoI $\underline{z}\left(t;\underline{\varphi }\right)$ using modal coordinates and mode shape vectors. Based on the modal expansion technique, the measured displacement or strain response time histories at $N_{0}$ degrees of freedom (DOFs) are given with respect to the modal coordinates by the mode superposition equation

(1) $$\underline{y}\left(t\right)=L\left(\underline{\delta }\right)\Phi \left(\underline{\varphi }\right)\underline{\xi }\left(t\right)+\underline{e}\left(t\right)$$

where $\underline{\xi }\left(t\right)\in R^{m}$ is the vector of *m* modal coordinates satisfying the modal equations, $L\left(\underline{\delta }\right)\in R^{N_{0}\times n}$ is the observation matrix that maps the displacements at all *n* model DOF to the measured displacement or strain quantities indicated by the sensor location vector $\underline{\delta }$, $\Phi \left(\underline{\varphi }\right)\in R^{n\times m}$ is the displacement mode shape matrix corresponding to the *n* model DOF and *m* contributing modes, and $\underline{e}\left(t\right)$ is a multi-variable zero-mean Gaussian noise term with covariance matrix $Q_{e}$ that accounts for measurement and model errors. The modal equation can be written as

(2) $$\ddot{\underline{\xi }}\left(t\right)+Z\left(\underline{\varphi }\right)\dot{\underline{\xi }}\left(t\right)+\Lambda \left(\underline{\varphi }\right)\underline{\xi }\left(t\right)=\Phi^{T}\left(\underline{\varphi }\right)M\underline{u}\left(t\right)$$

where Λ is the diagonal matrix of the squares $\omega_{r}^{2}$ of the modal frequencies $\omega_{r}$, *Z* is the diagonal matrix with the *r*-th diagonal element equal to $2\omega_{r}\zeta_{r}$, $\zeta_{r}$ is the modal damping ratio, and $M\in R^{n\times n_{u}}$ is a matrix of zeros and ones associating the independent excitations in the vector $\underline{u}\left(t\right)$ to the DOF of the structural model. Displacement, strain and/or stress predictions at output locations or DOF are given by the prediction equation

(3) $$\underline{z}\left(t\right)=\Psi \left(\underline{\varphi }\right)\underline{\xi }\left(t\right)+\underline{\varepsilon }\left(t\right)$$

where $\Psi \left(\underline{\varphi }\right)\in R^{n_{z}\times m}$ are the corresponding displacement, strain and/or stress mode shapes that relates the modal coordinates to displacement, strain and/or stress QoI, and $\underline{\varepsilon }\left(t\right)$ is a zero-mean prediction error with covariance matrix $Q_{\varepsilon }$ accounting for model error. The mode shape matrices $\Phi (\underline{\varphi })$ and $\Psi (\underline{\varphi })$ are available by analyzing the model (e.g., finite element model) of the structure.

It should be noted that formulation using Equations (1) and (3) can also be used when the available measurements and predictions consist of accelerations. In this case the modal vector $\underline{\xi }\left(t\right)$ in Equations (1) and (3) refers to the second derivatives of the modal coordinates with respect to time.

### 2.2. Bayesian Virtual Sensing

Bayesian inference is next used to estimate the modal vector parameter $\underline{\xi }\left(t\right)\equiv \underline{\xi }\left(t;\underline{\delta },\underline{\varphi }\right)$ and its uncertainties based on the data collected from a sensor configuration $\underline{\delta }$ and then propagate these uncertainties to predictions of output QoI $\underline{z}\left(t\right)\equiv \underline{z}\left(t;\underline{\delta },\underline{\varphi }\right)$. The posterior probability distribution function (PDF) $p\left(\underline{\xi }\left(t\right)|\underline{y}\left(t\right),\underline{\delta },\underline{\varphi }\right)$ quantifying the uncertainty in the modal coordinates $\underline{\xi }\left(t\right)$ at time *t*, given the data $\underline{y}\left(t\right)$, the sensor configuration $\underline{\delta }$ and the model parameters $\underline{\varphi }$, takes the form

(4) $$p\left(\underline{\xi }\left(t\right)|\underline{y}\left(t\right),\underline{\delta },\underline{\varphi }\right)\propto p\left(\underline{y}\left(t\right)|\underline{\xi }\left(t\right),\underline{\delta },\underline{\varphi }\right)p\left(\underline{\xi }\left(t\right)|\underline{\varphi }\right)$$

where, using the assumption that $\underline{e}\left(t\right)$ in Equation (1) follows a Gaussian distribution $N(\underline{e}\left(t\right)|\underline{0},Q_{e})$ with mean $\underline{0}$ and covariance $Q_{e}$, the likelihood takes the form

(5) $$p\left(\underline{y}\left(t\right)|\underline{\xi }\left(t\right),\underline{\delta },\underline{\varphi }\right)=N\left(\underline{y}\left(t\right)|L\left(\underline{\delta }\right)\Phi \left(\underline{\varphi }\right)\underline{\xi }\left(t\right),Q_{e}\right)$$

The prior PDF $p(\underline{\xi }\left(t\right)|\underline{\varphi })$ is postulated to be a zero-mean Gaussian $p\left(\underline{\xi }\left(t\right)|\underline{\varphi }\right)=N\left(\underline{\xi }\left(t\right)|\underline{0},S\right)$ with covariance matrix *S*. Substituting Equation (5) into Equation (4) it is straightforward to show that the output modal coordinates follow a multi-variable normal distribution [37,43]

(6) $$p\left(\underline{\xi }\left(t\right)|\underline{y}\left(t\right),\underline{\delta },\underline{\varphi }\right)=N\left(\underline{\xi }\left(t\right)|\hat{\underline{\xi }}\left(t;\underline{\delta },\underline{\varphi }\right),\Sigma_{\xi |D}\left(\underline{\delta },\underline{\varphi }\right)\right)$$

with mean

(7) $$\hat{\underline{\xi }}\left(t;\underline{\delta },\underline{\varphi }\right)={\left[\Phi^{T}\left(\underline{\varphi }\right)L^{T}\left(\underline{\delta }\right)Q_{e}^{-1}\left(\underline{\delta },\underline{\varphi }\right)L\left(\underline{\delta }\right)\Phi \left(\underline{\varphi }\right)+S^{-1}\right]}^{-1}\Phi^{T}\left(\underline{\varphi }\right)L^{T}\left(\underline{\delta }\right)Q_{e}^{-1}\left(\underline{\delta },\underline{\varphi }\right)\underline{y}\left(t\right)$$

and covariance matrix

(8) $$\Sigma_{\xi |D}\left(\underline{\delta },\underline{\varphi }\right)={\left[\Phi^{T}\left(\underline{\varphi }\right)L^{T}\left(\underline{\delta }\right)Q_{e}^{-1}\left(\underline{\delta },\underline{\varphi }\right)L\left(\underline{\delta }\right)\Phi \left(\underline{\varphi }\right)+S^{-1}\right]}^{-1}$$

Using Equations (3) and (6) to propagate uncertainty to output QoI $\underline{z}\left(t\right)$, it can be readily obtained that $\underline{z}\left(t\right)$ also follows a multivariable normal distribution

(9) $$p\left(\underline{z}\left(t\right)|\underline{y}\left(t\right),\underline{\delta },\underline{\varphi }\right)=N\left(\underline{z}\left(t\right)|\hat{\underline{z}}\left(t;\underline{\delta },\underline{\varphi }\right),\Sigma_{z|D}\left(\underline{\delta },\underline{\varphi }\right)\right)$$

with mean

(10) $$\hat{\underline{z}}\left(t;\underline{\delta },\underline{\varphi }\right)=\Psi \left(\underline{\varphi }\right)\hat{\underline{\xi }}\left(t;\underline{\delta },\underline{\varphi }\right)$$

that depends on the data, and covariance matrix $\Sigma_{z|D}\left(\underline{\delta },\underline{\varphi }\right)$ given by

(11) $$\Sigma_{z|D}\left(\underline{\delta },\underline{\varphi }\right)=\Psi \left(\underline{\varphi }\right){\left[\Phi^{T}\left(\underline{\varphi }\right)L^{T}\left(\underline{\delta }\right)Q_{e}^{-1}\left(\underline{\delta },\underline{\varphi }\right)L\left(\underline{\delta }\right)\Phi \left(\underline{\varphi }\right)+S^{-1}\right]}^{-1}\Psi^{T}\left(\underline{\varphi }\right)+Q_{\varepsilon }$$

In particular, the variance of the *i*-th element $z_{i}\left(t;\underline{\delta },\underline{\varphi }\right)$ of the response vector $\underline{z}\left(t;\underline{\delta },\underline{\varphi }\right)$ is given by the *i*-th diagonal element $\Sigma_{z_{i}|D}\left(\underline{\delta },\underline{\varphi }\right)$ of the covariance matrix $\Sigma_{z|D}\left(\underline{\delta },\underline{\varphi }\right)$ as follows

(12) $$\Sigma_{z_{i}|D}\left(\underline{\delta },\underline{\varphi }\right)=\psi_{i}^{T}\left(\underline{\varphi }\right){\left[\Phi^{T}\left(\underline{\varphi }\right)L^{T}\left(\underline{\delta }\right)Q_{e}^{-1}\left(\underline{\delta },\underline{\varphi }\right)L\left(\underline{\delta }\right)\Phi \left(\underline{\varphi }\right)+S^{-1}\right]}^{-1}\psi_{i}\left(\underline{\varphi }\right)+Q_{\varepsilon_{i}}$$

where $\psi_{i}\left(\underline{\varphi }\right)$ denotes the *i*-th column of the matrix $\Psi^{T}\left(\underline{\varphi }\right)$, and $Q_{\varepsilon_{i}}$ is the *i*-th diagonal element of the matrix $Q_{\varepsilon }$.

For a Gaussian prior PDF $N(\underline{\xi }\left(t\right)|\underline{0},\Sigma_{\xi })$ of the modal coordinate vector $\underline{\xi }\left(t\right)$, the prediction of the QoI $\underline{z}$ prior to the data can be readily obtained from Equation (3) to be Gaussian with mean zero and covariance matrix $\Sigma_{z}\left(\underline{\varphi }\right)=\Psi \left(\underline{\varphi }\right)\Sigma_{\xi }\left(\underline{\varphi }\right)\Psi^{T}\left(\underline{\varphi }\right)+Q_{\epsilon }$, where $\Sigma_{\xi }\left(\underline{\varphi }\right)=S$ is the covariance matrix corresponding to the assigned prior distribution. In particular, the variance of the *i*-th element $z_{i}\left(t;\underline{\varphi }\right)$ prior to the data is given by

(13) $$\Sigma_{z_{i}}\left(\underline{\varphi }\right)=\psi_{i}^{T}\left(\underline{\varphi }\right)S\psi_{i}\left(\underline{\varphi }\right)+Q_{\varepsilon_{i}}$$

The posterior and prior variances $\Sigma_{z_{i}|D}\left(\underline{\delta },\underline{\varphi }\right)$ in Equation (12) and $\Sigma_{z_{i}}\left(\underline{\varphi }\right)$ in Equation (13), described in terms of the parameters $\underline{\varphi }$ and the sensor locations $\underline{\delta }$, are the main quantities involved in the next section to solve the optimal sensor location problem for virtual sensing and response reconstruction. It is clear that these variances are independent of the measurements/data $\underline{y}\left(t\right)$ and depend only on the structural model parameters $\underline{\varphi }$, the model and measurement error covariances $Q_{e}$ and $Q_{\varepsilon }$, as well as the covariance matrix *S* of the prior probability distribution of the modal coordinates $\underline{\xi }\left(t\right)$. For practical convenience and without loss of generality, stationarity conditions are assumed, where the covariance matrices $Q_{e}$, $Q_{\epsilon }$ and *S* are independent of time *t*. As a results the posterior and prior variances defined in Equations (12) and (13) do not depend on time *t*. The parameters that define the model/prediction and measurement error covariances can be included in the parameter set $\underline{\varphi }$. A probability distribution $p(\underline{\varphi })$ can be postulated to quantify the uncertainties in the values of model and input characteristics involved in $\underline{\varphi }$.

Note that the matrix $\Phi^{T}\left(\underline{\varphi }\right)L^{T}\left(\underline{\delta }\right)Q_{e}^{-1}\left(\underline{\delta },\underline{\varphi }\right)L\left(\underline{\delta }\right)\Phi \left(\underline{\varphi }\right)$ in Equation (12) is nonsingular only if the number of sensors is greater or equal to the number of modes. Thus, for S=0, the condition $N_{0}\geq m$ should be met in order for the system to be identifiable [36]. The prior covariance matrix *S* is particularly important when the condition $N_{0}\geq m$ is not met. This matrix contributes subjective information from the prior that allows the inversion of the matrix appearing in the first term of Equation (11) for values of $N_{0}<m$.

## 3. Optimal Sensor Placement Formulation

### 3.1. Expected Utility Using Information Gain

Information theory is next combined with utility theory to measure the usefulness of a sensor configuration for reliable virtual sensing that is robust to modeling and measurement uncertainties. The objective is to select the sensor locations that maximize the information contained in the data for predicting with the least uncertainty the output response QoI at desirable locations. A measure of the information gain for estimating a response QoI $z_{i}$, given a set of data $\underline{y}$ and the model parameters $\underline{\varphi }$, is the KL-div [83] between the prior and posterior probability distribution of the output QoI $z_{i}$, defined for an experimental design $\underline{\delta }$ as

(14) $${\tilde{D}}_{i}\left(\underline{\delta },\underline{y},\underline{\varphi }\right)=\int_{Z_{i}}p\left(z_{i}|\underline{y},\underline{\delta },\underline{\varphi }\right)\ln\frac{p(z_{i}|\underline{y},\underline{\delta },\underline{\varphi })}{p(z_{i}|\underline{\varphi })}dz_{i}$$

For several output QoI included in the vector $\underline{z}$, the measure can be extended to the weighted average of the information gain for all possible output QoI, given as

(15) $$\tilde{D}\left(\underline{\delta },\underline{y},\underline{\varphi }\right)=\sum_{i=1}^{N_{z}}w_{i}{\tilde{D}}_{i}\left(\underline{\delta },\underline{y},\underline{\varphi }\right)$$

with $\sum_{i=1}^{N_{z}}w_{i}=1$, $w_{i}\geq 0$, where the values of the weight $w_{i}$ are selected to quantify the importance of the *i*-th QoI $z_{i}$ in the design of the sensor configuration.

In the initial design phase the data are not available. Instead they can be generated by the prediction error model Equation (1) for given values of the model parameters $\underline{\varphi }$ and the probability distribution of the prediction error term $\underline{e}\left(t\right)$. Following Lindley’s work [68], utility theory is used to measure the usefulness of the experiment with the utility function selected to be the expected value of the information gain in Equation (15) over all possible values of the experimental data. Extending the utility function to include the uncertainty in the model parameters $\underline{\varphi }$ as well, one introduces the expected utility function

(16) $$U\left(\underline{\delta }\right)=\int_{\tilde{\Phi }}\int_{Y}\tilde{D}\left(\underline{\delta },\underline{\varphi },\underline{y}\right)p\left(\underline{y},\underline{\varphi }|\underline{\delta }\right)d\underline{y}d\underline{\varphi }=\sum_{i=1}^{N_{z}}w_{i}U_{i}\left(\underline{\delta }\right)$$

that quantifies the usefulness of learning from the data for predicting the output QoI included in the vector $\underline{z}$, in the presence of model and measurement uncertainties, where

(17) $$U_{i}\left(\underline{\delta }\right)=\int_{\tilde{\Phi }}\int_{Y}{\tilde{D}}_{i}\left(\underline{\delta },\underline{\varphi },\underline{y}\right)p\left(\underline{y},\underline{\varphi }|\underline{\delta }\right)d\underline{y}d\underline{\varphi }$$

is the expected utility function that accounts for a component $z_{i}$ of the response QoI $\underline{z}$, $p\left(\underline{y},\underline{\varphi }|\underline{\delta }\right)=p\left(\underline{y}|\underline{\varphi },\underline{\delta }\right)p\left(\underline{\varphi }\right)$, $p(\underline{y}|\underline{\varphi },\underline{\delta })$ is the uncertainty in the outcome $\underline{y}$ given the model parameters $\underline{\varphi }$, and $p(\underline{\varphi })$ is the uncertainty in the model parameters. The utility function defined in Equations (16) and (17) is an average of the information gain over all the possible data outcomes.

It is shown in Appendix A that the expected utility function $U_{i}\left(\underline{\delta }\right)$ can be formulated in terms of the change in the expected information entropy before and after the data are collected, given by

(18) $$U_{i}\left(\underline{\delta }\right)=\int_{\Phi }H_{z_{i}}\left(\underline{\varphi }\right)p\left(\underline{\varphi }\right)d\underline{\varphi }-\int_{\tilde{\Phi }}\int_{Y}H_{z_{i}|D}\left(\underline{\delta },\underline{y},\underline{\varphi }\right)p\left(\underline{y}|\underline{\varphi },\underline{\delta }\right)d\underline{y}p\left(\underline{\varphi }\right)d\underline{\varphi }$$

where $H_{z_{i}}\left(\underline{\varphi }\right)$ is the prior information entropy in $z_{i}\left(t\right)$ given the model parameter set $\underline{\varphi }$, and $H_{z_{i}|D}\left(\underline{\delta },\underline{y},\underline{\varphi }\right)$ is the posterior information entropy in $z_{i}\left(t\right)$ given the data $\underline{y}$ and the model parameter set $\underline{\varphi }$. For Gaussian probability distribution of the response $z_{i}\left(t\right)$, the posterior information entropy given the values of the data set and the model parameter set $\underline{\varphi }$ is given in terms of the *i*-th diagonal component of the covariance matrix $\Sigma_{z|D}\left(\underline{\delta },\underline{\varphi }\right)$ of the error in the estimate of $\underline{z}$ as follows

(19) $$H_{z_{i}|D}\left(\underline{\delta },\underline{y},\underline{\varphi }\right)\equiv H_{z_{i}|D}\left(\underline{\delta },\underline{\varphi }\right)=\frac{1}{2}\left[\ln\left(2\pi \right)+1\right]+\frac{1}{2}\ln\det\Sigma_{z_{i}|D}\left(\underline{\delta },\underline{\varphi }\right)$$

Thus it depends on the sensor locations and the values of the parameters set $\underline{\varphi }$, while it is independent of the data. For the prior information entropy $H_{z_{i}}\left(\underline{\varphi }\right)$ an expression similar to Equation (19) holds with the posterior $\Sigma_{z_{i}|D}\left(\underline{\delta },\underline{\varphi }\right)$ replaced by the prior $\Sigma_{z_{i}}\left(\underline{\varphi }\right)$.

Taking into account that the prior information entropy $H_{z_{i}}\left(\underline{\varphi }\right)$ in Equation (18) is constant, independent of the sensor configuration $\underline{\delta }$, and that the posterior information entropy $H_{z|D}\left(\underline{\delta },\underline{y},\underline{\varphi }\right)$ does not depend on the data, the expected utility function $U_{i}$ finally takes the form

(20) $$U_{i}\left(\underline{\delta }\right)=-\Delta {\bar{H}}_{i}\left(\underline{\delta }\right)=-\left[{\bar{H}}_{z_{i}|D}\left(\underline{\delta }\right)-{\bar{H}}_{z_{i}}\right]=-\frac{1}{2}\int_{\tilde{\Phi }}\ln\frac{\Sigma_{z_{i}|D}\left(\underline{\delta },\underline{\varphi }\right)}{\Sigma_{z_{i}}\left(\underline{\varphi }\right)}p\left(\underline{\varphi }\right)d\underline{\varphi }$$

where

(21) $${\bar{H}}_{z_{i}|D}\left(\underline{\delta }\right)=\int_{\tilde{\Phi }}H_{z_{i}|D}\left(\underline{\delta },\underline{\varphi }\right)p\left(\underline{\varphi }\right)d\underline{\varphi }$$

and

(22) $${\bar{H}}_{z_{i}}=\int_{\tilde{\Phi }}H_{z_{i}}\left(\underline{\varphi }\right)p\left(\underline{\varphi }\right)d\underline{\varphi }$$

are respectively the expected posterior and prior information entropies over all possible values of the model parameters $\underline{\varphi }$, weighted by the PDF $p(\underline{\varphi })$ of the model parameters.

Substituting Equation (20) into Equation (16), the expected utility function that accounts for all response entries in the vector $\underline{z}$ takes the form

(23) $$U\left(\underline{\delta }\right)=-\Delta \bar{H}\left(\underline{\delta }\right)=-\sum_{i=1}^{n_{z}}w_{i}\Delta {\bar{H}}_{i}\left(\underline{\delta }\right)=-\frac{1}{2}\sum_{i=1}^{n_{z}}w_{i}\int_{\Phi }\ln r\left(\underline{\delta },\underline{\varphi }\right)p\left(\underline{\varphi }\right)d\underline{\varphi }$$

where $r(\underline{\delta },\underline{\varphi })$ is defined as the ratio

(24) $$r\left(\underline{\delta },\underline{\varphi }\right)=\frac{\Sigma_{z_{i}|D}\left(\underline{\delta },\underline{\varphi }\right)}{\Sigma_{z_{i}}\left(\underline{\varphi }\right)}$$

Using equal weight values $w_{i}=1/n_{z}$, the utility function takes the form

(25) $$U\left(\underline{\delta }\right)=-\frac{1}{2n_{z}}\int_{\tilde{\Phi }}\ln\frac{\prod_{i=1}^{n_{z}}\left[\Sigma_{z_{i}|D}\left(\underline{\delta },\underline{\varphi }\right)\right]}{\prod_{i=1}^{n_{z}}\left[\Sigma_{z_{i}}\left(\underline{\varphi }\right)\right]}p\left(\underline{\varphi }\right)d\underline{\varphi }$$

The integral in Equation (23) or Equation (25) is a probability integral over the space of uncertain parameters $\underline{\varphi }$. This integral represents the robust information entropy change before and after the data are available, weighted over all possible values of the model parameters quantified by the PDF $p(\underline{\varphi })$. The multidimensional integral can be evaluated using Monte Carlo techniques or sparse grid methods [85,86]. It is verified in Appendix B that the ratio $r(\underline{\delta },\underline{\varphi })$ in Equation (24) cannot exceed the value of 1 and so the information entropy change is always non-positive or, equivalently the utility function is non-negative as expected, meaning that there can be only information gain when placing a given number of sensors in the structure.

### 3.2. Optimal Sensor Placement

The optimal sensor configuration ${\underline{\delta }}_{opt}$ is obtained by maximizing the utility $U\left(\underline{\delta }\right)$ or, equivalently, minimizing the change in information entropy $\Delta {\bar{H}}_{z|D}\left(\underline{\delta }\right)$, with respect to the design variables $\underline{\delta }$, that is

(26) $${\underline{\delta }}_{opt}=\arg_{\underline{\delta }}\max U\left(\underline{\delta }\right)=\arg_{\underline{\delta }}\min\Delta {\bar{H}}_{z|D}\left(\underline{\delta }\right)$$

The optimal number of sensors in the sensor configuration can be estimated by monitoring the gain in information as additional sensors are placed in the structure. Usually, after sufficiently number of sensors are placed in the structure, the information gain using additional sensors is relatively small and the process of adding sensors in the structure is terminated.

The optimization in Equation (26) may result in multiple local/global solutions [57]. The optimization problem can be solved using continuous design variables $\underline{\delta }$ accounting for the location of the sensors over the physical domain of the structure or discrete design variables $\underline{\delta }$ accounting for the discrete locations (e.g., DOF at nodes for placing displacement/acceleration sensors or Gauss integration points for placing strains sensors in a finite element mesh). Global optimization algorithms [87,88] as well as stochastic optimization algorithms, such as CMA-ES [89] and genetic algorithms [45,90,91,92,93] can be employed in order to avoid premature convergence to a local optimum. Alternative heuristic forward and backward sequential sensor placement (FSSP/BSSP) algorithms [54,57] are effective in solving the optimization problem. The heuristic algorithms bypass the problem of multiple local/global optima manifested in optimal experimental designs, providing near optima solutions in a fraction of the computational effort required in stochastic optimization algorithms or exhaustive search methods [52]. For a total of $N_{all}$ possible sensors positions and $N_{0}$ sensors to be placed in the structure, for $N_{0}$ relatively small compared to $N_{all}$ the FSSP algorithm requires approximately $N_{F}=N_{0}N_{all}$ function evaluations, while the BSSP algorithm requires approximately $N_{B}=N_{all}\left(N_{all}+1\right)/2$ function evaluations [54]. Although the computational effort for the BSSP is approximately $N_{B}/N_{F}\approx 0.5N_{all}/N_{0}$ times larger than the computational effort of FSSP and thus FSSP should be preferred for $N_{0}<<N_{all}$, the estimate from BSSP may in some cases be better than the FSSP estimate and thus the combined estimate should be used in the optimization. More details are presented in the applications Section 6.

## 4. Model Prediction Error Formulation

Following the analysis in Section 2.1, the prediction error $\underline{e}={\underline{e}}_{meas}+{\underline{e}}_{model}$ in Equation (1) is partly due to a term, ${\underline{e}}_{meas}$, accounting for the measurement error and partly due to a term, ${\underline{e}}_{model}$, accounting for the model error. Assuming that measurement and model errors are independent and zero-mean Gaussian vectors with covariance matrices $Q_{e,meas}$ and $Q_{e,model}$, the covariance of the total prediction error is

(27) $$Q_{e}=Q_{e,meas}+Q_{e,model}$$

To proceed with the optimal sensor placement design one has to select the values of the covariance matrices $Q_{e,meas}$ and $Q_{e,model}$. The selection depends on the nature of the problem analyzed. The following selections follow the suggestions presented in [94]. For the measurement error term it is reasonable to assume independence of the values of the errors from the intensity of the response so that $Q_{e,meas}=s^{2}I$, where *I* is the identity matrix, and the level *s* depends on the sensor accuracy and characteristics. A reasonable choice of the model error variance $Q_{e,model}^{\left(ii\right)}$ at the *i*-th DOF is to have it proportional to the square of the intensity of the QoI at DOF *i*, given by $Q_{y}^{\left(ii\right)}$. That is, we select $Q_{e,model}^{\left(ii\right)}=\sigma_{e}^{2}Q_{y}^{\left(ii\right)}$, where $\sigma_{e}$ denotes the level of model error in relation to the intensity of the QoI. In this way, the level of model error is independent on the intensity of the response. In addition, a certain degree of correlation is expected for the model errors between two neighborhood locations, arising from the underlining model dynamics [57]. This correlation can be taken into account by selecting a non-diagonal covariance matrix $Q_{e,model}$. The correlation $Q_{e,model}^{\left(ij\right)}$ between the predictions errors $e_{i,model}$ and $e_{j,model}$ at DOFs *i* and *j*, respectively, can be assumed to be

(28) $$Q_{e,model}^{\left(ij\right)}=\sqrt{Q_{e,model}^{\left(ii\right)}Q_{e,model}^{\left(jj\right)}}\rho \left(d_{ij}\right)$$

so that it accounts for the spatial distance $d_{ij}$ between the DOFs *i* and *j*, where $\rho (d_{ij})$ is a correlation function satisfying ρ(0)=1. However, in the experimental design phase where data are not available, the actual errors and correlations should be postulated in order to proceed with the design of the optimal sensor locations. Several correlation functions can be explored. For demonstration purposes in this study, the following exponentially decaying correlation function is assumed:

(29) $$\rho \left(d_{ij}\right)=exp\left(-\frac{d_{ij}}{\lambda }\right)$$

where λ is a measure of the spatial correlation length. However, the formulation in this work is general and does not depend on the choice of the correlation model.

Using the aforementioned selections, the covariance matrix $Q_{e}$ in Equation (27), required in Equation (12), simplifies to

(30) $$Q_{e}=s^{2}I+\sigma_{e}^{2}{\tilde{Q}}_{y}^{1/2}R{\tilde{Q}}_{y}^{1/2}$$

where the notation $\tilde{Q}$ denotes a diagonal matrix that contains in *i*-th diagonal entry the quantity $Q_{y}^{\left(ii\right)}$, ${\tilde{Q}}^{1/2}$ represents a diagonal matrix with elements the square roots of the elements in $\tilde{Q}$, and *R* is the correlation matrix with the (i,j) element $R_{ij}$ equal to $\rho (d_{ij})$.

A similar formulation can be used for the variance $Q_{\epsilon_{i}}$ involved in Equation (12) of the error ${\underline{\varepsilon }}_{i}$ for the predicted QoI $z_{i}$. In this case only the model error exists and the *i*-th diagonal element of the covariance matrix can be selected to be

(31) $$Q_{\varepsilon_{i}}=\sigma_{\varepsilon }^{2}Q_{z_{i}}$$

were $Q_{z_{i}}$ is the square of the intensity of the predicted QoI $z_{i}\left(t\right)$, and $\sigma_{\varepsilon }$ is the level of model error in relation to the intensity of the predicted QoI.

The formulation for the errors and the mathematical structure of the ratio $r(\underline{\theta },\underline{\varphi })$ in Equation (24) can be used to show a number of useful properties for the utility function and thus for the information gain. Specifically, in Appendix B.1 it is shown that as the measurement and model/prediction errors increase for a given sensor configuration, the ratio $r(\underline{\theta },\underline{\varphi })$ increases and so the utility function decreases. This indicates that the higher the errors, the less the information gain from the sensor configuration. Finally, another important property shown in Appendix B.2 is that adding a sensor in an existing sensor configuration increases the information gain, which is similar to the results presented in [54] for parameter estimation. This should be expected since as a sensor is added in an existing sensor configuration, there can be null or extra information provided by this sensor. As a result, the maximum and minimum value of the utility function is an increasing function of the number of sensors. Lastly, the spatially correlated structure of the model error, introduced in Equation (29), has the important effect of avoiding clustering of sensors as it was theoretically shown in [57] for OSP for parameter estimation.

## 5. Implementation

It should be noted that the estimate in Equation (10) of the response QoI $\underline{z}$ and the error in the estimate, quantified by the covariance matrix $\Sigma_{z}\left(\underline{\delta },\underline{\varphi }\right)$ in Equation (12), depends on the measurement/model and prediction error covariance matrices $Q_{e}$ and $Q_{\epsilon }$, as well as the covariance matrix *S* of the prior Normal distribution assumed for the modal coordinates $\underline{\xi }\left(t\right)$. The effect of the values of these covariance matrices on the optimal sensor placement for response predictions is investigated in this work.

The selection of the the covariance matrix *S* of the prior normal distribution should take into account the relative contribution of the different modal coordinates on the response of the system. Such contribution depends highly on the excitation characteristics. Equations (30) and (31) also suggest that the values of the covariance matrices $Q_{e}$ and $Q_{\varepsilon }$ of the model prediction errors should be carefully selected based on the intensity of the measured and predicted responses which are not known at the OSP design phase. To proceed with rational selections, the intensities of measured and predicted QoI have to be considered which depend on the characteristics of the excitation. Thus, the characteristics of the excitation have to be considered in the analysis in order to decide on the values of the covariance matrices *S*, $Q_{e}$ and $Q_{\epsilon }$ upon which the OSP design will be based. Failing to consider the intensity of the modal coordinates and the responses in the selection of the prior and error covariance matrices may lead to OSP designs that are based on non-rational choices of these error covariance matrices.

Due to the uncertainty in the excitation characteristics the values to be assigned for the model/prediction and measurement errors involve large uncertainty. We proceed with a thorough investigation of the effect of the model/prediction and measurement errors as well as the effect of the uncertainty in the prior distribution on the information gain and the optimal sensor location. Finally, the robust design proposed in this work will take into account these uncertainties in the design of the optimal sensor configuration.

To demonstrate concepts, we assume a zero-mean stationary white noise excitation. We also assume, without loss of generality, that the location of the excitation is known. Unknown locations or multiple excitation components can as well be treated in the formulation. However, such an analysis is beyond the scope of the present work. Using a linear model (e.g., a finite element model) of the structure, one can readily obtain the covariance $Q_{\xi }$ of the modal quantities, as well as the covariance of the response QoI (displacements, velocities, strains and stresses) $Q_{y}$ and $Q_{z}$. These matrices can be used in Equations (30) and (31) to make the proper assignment for $Q_{e}$ and $Q_{\varepsilon }$ through the proper selection of the prediction error parameters $\sigma_{e}$, $\sigma_{\varepsilon }$ and *s*. Furthermore, accepting that the excitation is white noise, it is also reasonable to assume that the covariance of the prior distribution for $\underline{\xi }\left(t\right)$ is selected to be proportional to the covariance $Q_{\xi }$ of the modal quantities $\underline{\xi }\left(t\right)$, i.e.,

(32) $$S=\alpha^{2}Q_{\xi }$$

where α quantifies the extent of the uncertainty in the prior distribution. This assignment will correctly take into account the participation of each mode in the vibration analysis of the structure.

The analysis for estimating the covariance matrix $Q_{\xi }$ of the modal coordinates and the covariance matrices $Q_{y}$ and $Q_{z}$ of various response QoI is presented next based on the finite element model of the structure and a discrete state space representation of the modal equations in Equation (2). Introducing the state space vector ${\underline{x}}_{k}^{T}={\left[\begin{array}{cc} {\underline{\xi }}_{k}^{T} & {\dot{\underline{\xi }}}_{k}^{T} \end{array}\right]}^{T}$ at time instant t=kΔt, where ${\underline{\xi }}_{k}=\underline{\xi }\left(k\Delta t\right)$ and Δt is the sampling time of a signal, the modal equation in Equation (2) can be written in the discrete state space form

(33) $${\underline{x}}_{k+1}=A{\underline{x}}_{k}+B{\underline{u}}_{k}$$

where, using zero-order hold, the state space matrices are given as $A=exp(A_{c}\Delta t)$ and $B=\left(A-I\right)A_{c}^{-1}B_{c}$ with

(34) $$A_{c}=\left[\begin{array}{cc} 0 & I\\ -\Lambda & -Z \end{array}\right],\;B_{c}=\left[\begin{array}{c} 0\\ \Phi^{T}M \end{array}\right]$$

An output vector QoI ${\underline{h}}_{k}$ at time t=kΔt, either it corresponds to measured quantities $\underline{y}$ or predicted quantities $\underline{z}$, can be written in the form

(35) $${\underline{h}}_{k}=C{\underline{x}}_{k}+D{\underline{u}}_{k}$$

where the matrices *C* and *D* relate the response QoI to the state vector and input load vector, respectively. For displacement, strain or stress responses at all DOF of the structure, the matrices $C=G\Phi [\begin{array}{cc} I & 0 \end{array}]$ and D=0, where the matrix *G* relates the displacement DOF with the output QoI (displacements, strains and stresses). For acceleration responses the matrices $C=G\Phi [\begin{array}{cc} -\Lambda & -Z \end{array}]$ and $D=G\Phi \Phi^{T}M$. Here it is assumed that Φ is mass normalized. In particular, to estimate ${\underline{\ddot{\xi }}}_{k}$ one uses Equation (35) with $C=[\begin{array}{cc} -\Lambda & -Z \end{array}]$ and $D=\Phi^{T}M$.

Assuming a scalar stationary zero-mean Gaussian white noise excitation with variance $\sigma_{wn}^{2}$, the covariance $Q_{x}$ of the state vector under stationary conditions is given by $Q_{x}=\sigma_{wn}^{2}{\bar{Q}}_{x}$, where ${\bar{Q}}_{x}$ can be obtained by solving the discrete Liapunov equation

(36) $$A{\bar{Q}}_{x}A^{T}-{\bar{Q}}_{x}+BB^{T}=0$$

Using Equation (35), the covariance of the output response QoI in the vector $\underline{h}\left(t\right)$ is given by

(37) $$Q_{h}=\sigma_{wn}^{2}\left(C{\bar{Q}}_{x}C^{T}+DD^{T}\right)$$

and is proportional to the variance $\sigma_{wn}^{2}$ of the discrete white noise excitation. Setting $\underline{h}=\underline{y}$, or $\underline{h}=\underline{z}$, or $\underline{h}=\underline{\xi }$, the covariance matrices $Q_{y}$, or $Q_{z}$, or $Q_{\xi }$ are obtained, required in the error covariance matrices $Q_{e}$ and $Q_{\varepsilon_{i}}$ in Equations (30) and (31) and the prior distribution *S* in Equation (32).

## 6. Applications

**Figure 1:**
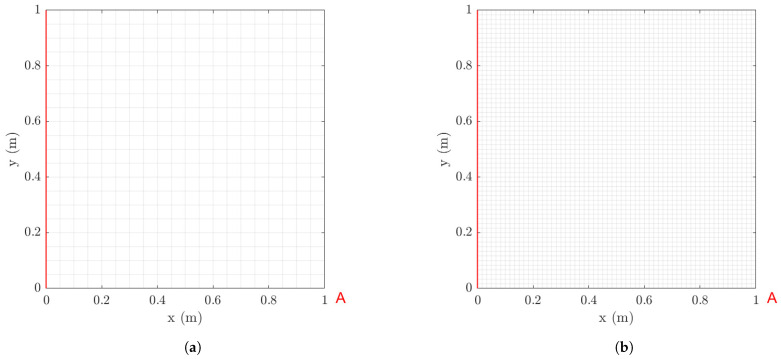
Square thin plate with left edge fixed (shown in red line); (**a**) coarse mesh, (**b**) fine mesh.

The methodology is demonstrated for a square plate structure modeled by thin-shell finite elements (FEs). The plate is fixed at the left edge. The model is meshed with eight-node shell elements containing six DOFs per node (Figure 1). To investigate the effect of mesh size on the optimal sensor placement, two models are considered corresponding to different mesh types: a coarse and a fine mesh. The coarse mesh model consist of 420 elements, 441 nodes, while the fine mesh model consists of 3660 elements and 3721 nodes. Linear elastic behavior is assumed. The lowest eight natural frequencies of the models for the coarse and fine mesh are presented in Table 1.

### 6.1. Strain Predictions Using Strain Observations

Normal strain measurements and predictions are considered along the *x* direction at the midpoints of all finite elements of the plate surface comprising the mesh. OSP of strain sensors is performed for predicting the strains at all finite elements of the mesh (Figure 1). Contribution of the lower eight modes to the dynamic behavior of the plate is assumed in designing the optimal location of sensors.

#### 6.1.1. Model/Prediction Errors, Measurement Error and Prior Distribution

**Figure 2:**
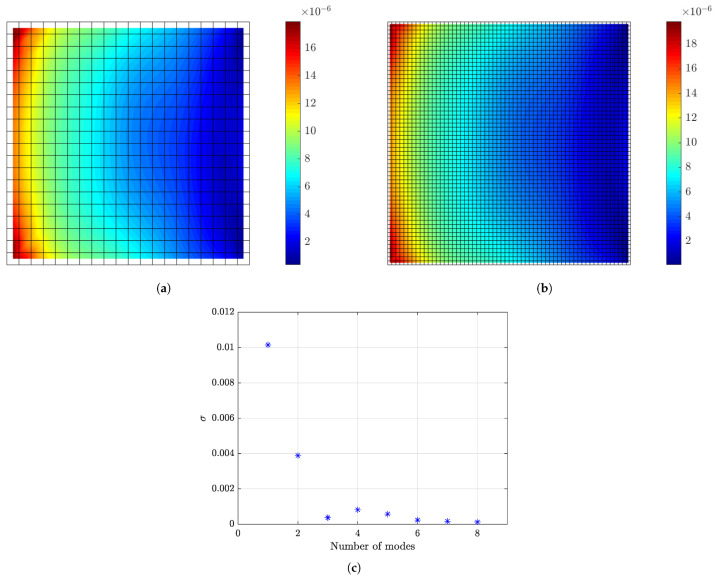
Intensities of normal strains computed along the x direction at the middle of the finite elements of the mesh (**a**) for coarse mesh, (**b**) for fine mesh. (**c**) Intensities of the modal coordinates $Q_{\xi }^{1/2}$ as a function of the number of modes.

Reasonable choices of the error parameters *s*, $\sigma_{e}$ and $\sigma_{\varepsilon }$ involved in the covariance matrices $Q_{e}$ and $Q_{\varepsilon }$ in Equations (30) and (31) of the model/prediction and measurement error models are next considered. For this, it is assumed that the plate is subjected to a concentrated load applied at the right bottom corner A, as shown in Figure 1. A broad band excitation is considered, modeled by a discrete Gaussian white noise sequence with standard deviation $\sigma_{wn}$. To select the standard deviation *s* of the measurement error, the intensities of the normal strain responses along the *x* direction predicted for white noise input are computed and shown in Figure 2a,b for the coarse and fine mesh, respectively. The intensity of a response QoI $z_{i}$ is quantified by the standard deviation $Q_{z_{i}}^{1/2}$ computed by solving the Liapunov Equation (36) and using Equation (37). The results in Figure 2 are normalized with respect to the intensity $\sigma_{wn}$ of the white noise input. Approximately 98% of the computed intensities of the strains in all plate elements are greater than $\epsilon_{min}\equiv Q_{z,min}^{1/2}=10^{-6}$, while the maximum strain intensity value is approximately $\epsilon_{max}\equiv Q_{z,max}^{1/2}=2\times 10^{-5}=20\epsilon_{min}$. To investigate the effect of measurement error, the parameter *s* of the error covariance matrix in Equation (30) is selected as shown in Table 2 to have four different values corresponding to very small, small, moderate and large measurement error, respectively. The $\sigma_{e}$ of the model error and $\sigma_{\varepsilon }$ of the prediction error involved in Equations (30) and (31) are selected to be $\sigma_{e}=\sigma_{\varepsilon }=0.01$ and 0.001 corresponding to small and very small model/prediction errors, respectively. The case of uncorrelated prediction error is considered (λ=0 in Equation (29)).

The intensities of the modal coordinates $\underline{\xi }\left(t\right)$ predicted for white noise input are shown in Figure 2c. It is clear that the intensities of the modal coordinates vary considerably from mode to mode. This reinforces the fact that the covariance of the prior distribution of the modal coordinates should carefully be chosen using Equation (32) to take into account the different intensities of each mode. These modal intensities highly depend on the spatial and temporal excitation characteristics (number and location of excitation, frequency characteristics, etc.). The value of α in Equation (32) is selected to be $\alpha =10^{2}$ and α=1 corresponding respectively to large and small uncertainties in the prior distribution of the modal coordinates $\underline{\xi }$.

#### 6.1.2. FSSP and BSSP Algorithms

**Figure 3:**
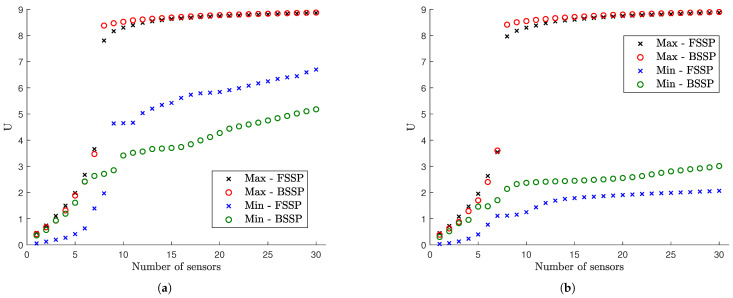
Comparison of the maximum and minimum utility values as a function of the number of sensors obtained from FSSP and BSSP algorithms for (**a**) coarse mesh and (**b**) fine mesh.

The results for the utility values obtained using the FSSP and BSSP algorithms for $\sigma_{e}=\sigma_{\varepsilon }=0.01$ (small model/prediction errors), $s=10^{-7}$ (moderate measurement error) and $\alpha =10^{2}$ (large prior uncertainty in modal coordinates) are compared in Figure 3 for coarse (Figure 3a) and fine meshes (Figure 3b). The estimates from the two algorithms differ due to the fact that both algorithms are heuristic and provide approximate values. In this specific case and for the coarse mesh, the BSSP algorithm provides better solutions for the maximum and minimum utility for more than eight sensors, while the FSSP algorithm provides better solution than the BSSP algorithm for one to seven sensors. This observation is not consistent for the fine mesh where FSSP algorithms provides better estimates for the minimum utility for all number of sensors, while the BSSP provides a better estimate for the maximum utility for seven sensors. Similar behavior for the accuracy of the results provided from the FSSP and BSSP algorithms is observed for other error cases as well.

To increase the reliability of the estimates arising from the two heuristic algorithms, the final solution is taken from the combination of the FSSP and BSSP solutions. Specifically, for each sensor configuration containing a fixed number of sensors, the final maximum utility value is taken to be $U_{max}=max\left(U_{F,max},U_{B,max}\right)$, where $U_{F,max}$ and $U_{B,max}$ are the maximum values estimated from the FSSP and BSSP algorithms, respectively. Additionally, the optimal sensor placement is selected among the FSSP and BSSP optimal sensor placement that corresponds to the value of $U_{max}$. A similar procedure is used for the minimum utility value, i.e., $U_{min}=max\left(U_{F,min},U_{B,min}\right)$. The combined FSSP/BSSP result will be referred from here on as the sequential sensor placement (SSP) estimate.

The use of BSSP to obtain results has the effect of raising substantially the computational cost in relation to FSSP. Specifically, comparing the number of function evaluations $N_{F}$ and $N_{B}$ for the FSSP and BSSP algorithms one has that for $N_{0}=30$ sensors that $N_{B}/N_{F}=0.5N_{all}/N_{0}\approx 6$ for the coarse mesh and $N_{B}/N_{F}\approx 60$ for the fine mesh. The number of functions evaluations for BSSP for the fine mesh is two order of magnitude larger than the one required for FSSP. Additionally, for the spatially correlated prediction error case, the FSSP and BSSP require the repeated solutions of algebraic linear system of equations of size (see Equation (12)) as high as $N_{0}$ and $N_{all}$, respectively, raising substantially the computational effort for BSSP in relation to FSSP for the common case for which the number of possible sensor locations $N_{all}$ is usually much higher than number of sensors $N_{0}$ ($N_{all}>>N_{0}$) in a sensor configuration.

#### 6.1.3. Information Gain versus Number of Sensors

**Figure 4:**
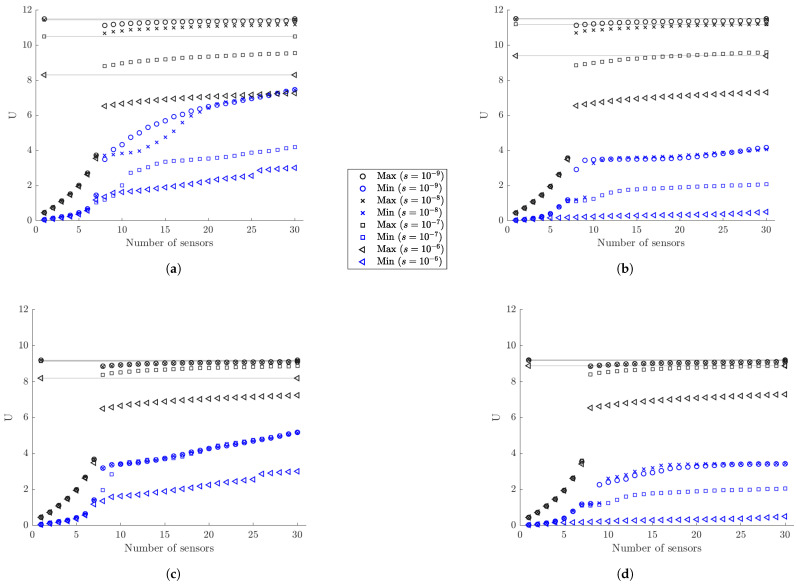
SSP results for maximum and minimum utility values for model/prediction error $\sigma_{e}=\sigma_{\varepsilon }=0.001$ (**a**,**b**) and $\sigma_{e}=\sigma_{\varepsilon }=0.01$ (**c**,**d**) obtained for the coarse (**a**,**c**) and fine (**b**,**d**) meshes. Results are presented for different measurement errors s as shown in the legend and for $\alpha =10^{2}$. The horizontal lines are the maximum utility values that can be achieved by using strain sensors at all finite elements of the coarse and fine meshes.

The SSP results for the maximum and minimum utility values as a function of the number of sensors for the optimal and worst sensor configurations for up to 30 sensors are shown in Figure 4 for different measurement and model/prediction errors for both coarse (Figure 4a,c) and fine (Figure 4b,d) meshes. Large prior uncertainty in the modal coordinates is assumed ($\alpha =10^{2}$). Comparing the maximum utility values in Figure 4a,c for the coarse mesh with the corresponding maximum utility values in Figure 4b,d for the fine mesh, it can be seen that the results are almost indistinguishable. Thus, the mesh size does not affect the maximum value of the expected information gain, as it should be expected since the dynamic characteristics from both meshes do not differ significantly as shown in Table 1. However, the mesh size affects the minimum value of the information gain, providing substantially lower values of the utility for the fine mesh. This is due to the fact that a fine mesh contains significantly more finite elements and thus more strain sensor locations with non-informative strains than the coarse mesh does. It should be noted that the difference between maximum and minimum expected information entropy values for a fixed number of sensors gives the maximum information gain that can be achieved by employing the optimal sensor placement methodology.

To interpret the results in Figure 4, it should be kept in mind that for eight contributing modes one needs at least eight sensors in order for the information matrix in Equation (12) to be invertible and the problem to be identifiable without the use of the subjective information from the prior PDF of the modal coordinates. For less than eight sensors the information matrix in Equation (12) is not invertible without prior information. The prior covariance matrix *S* of the modal coordinates provides the missing information required to make the problem identifiable. From the results in Figure 4a, it is observed that the expected information gain steadily increases as one adds from one to seven sensors due to the increase of the information from the data, and rises sharply from seven to eight sensors due to the fact that eight sensors placed at their optimal positions provide the necessary information without the need of the small complementary information from the prior.

**Figure 5:**
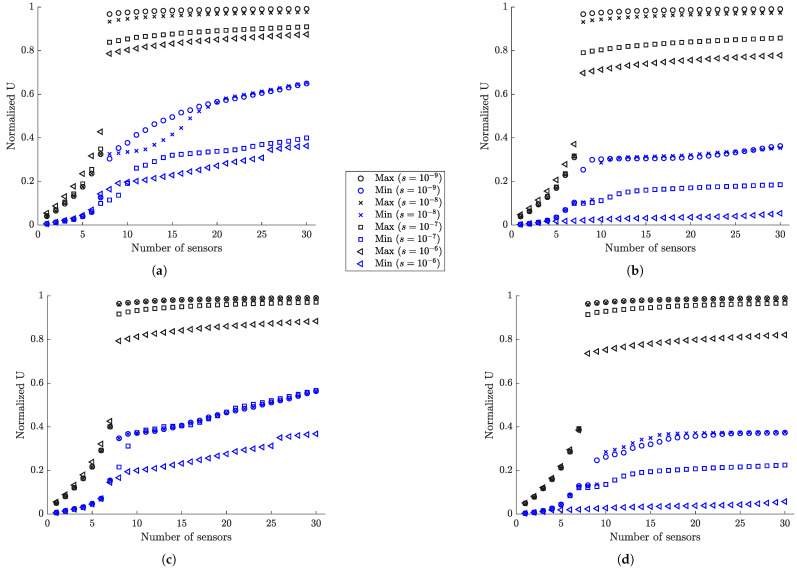
Normalized utility values for model/prediction error $\sigma_{e}=\sigma_{\varepsilon }=0.001$ (**a**,**b**) and $\sigma_{e}=\sigma_{\varepsilon }=0.01$ (**c**,**d**) obtained for the coarse (**a**,**c**) and fine (**b**,**d**) meshes. Results are presented for different measurement errors s as shown in the legend and for $\alpha =10^{2}$.

Normalized utility values obtained by dividing the maximum and minimum utility values in Figure 4 by the utility values obtained by placing the maximum number of strain sensors at all finite elements of the mesh (420 for coarse mesh and 3660 for fine mesh) are presented in Figure 5 for each measurement and model/prediction error case. By tracking the maximum normalized information gain values as a function of the number of sensors, it is possible to decide on the number of sensors to be kept in an optimal sensor configuration. One should stop adding sensors in the structure when the information gained by additional sensors is not significant compared to the information gained by the existing sensors, or when the information gained by a number of sensors is a sufficiently large percentage of the maximum information that can be achieved by placing sensors at all possible locations (e.g., all finite elements of the mesh in the plate problem).

#### 6.1.4. Information Gain versus Measurement Error

As seen in Figure 5a,b, for very small model error ($\sigma_{e}=\sigma_{\epsilon }=0.001$) and for very small ($s=10^{-9}$) to small ($s=10^{-8}$) measurement error, eight sensors placed at their optimal positions account for approximately 97% to 93% of the maximum information that can be gained by adding strain sensors at all possible locations. For moderate ($s=10^{-7}$) and large ($s=10^{-6}$) measurement error, eight optimally located sensors provide an information gain of the order of 84% and 79% for the coarse mesh and 79% and 70% for the fine mesh compared to the maximum information gain that can be achieved for the coarse and fine mesh, respectively. These lower values are due to the fact that information extracted from sensors is affected by the model and measurement errors. The higher the error, the less the information extracted from the sensors. Additionally, comparing the normalized information gain values for the fine and coarse meshes, smaller normalized information gain values are reported for the fine mesh due to the fact that in these large error case the 3660 strain sensors provide more information than the 440 strain sensors placed at all finite element of the fine and coarse mesh, respectively. Thus the normalizing quantity for the fine mesh is highest for the fine mesh and as a result the normalized information gain values for the fine mesh appear smaller than the corresponding ones for the coarse mesh.

For the small measurement error cases ($s=10^{-9}$ and $s=10^{-8}$), eight sensors placed at their optimal positions provide most of the information for accurate response prediction (Figure 5). Given that eight sensors have been placed on the structure, there is very small gain in information (less than 7%) if the plate is fully populated with sensors. This is mainly due to the fact that quality of the measurements is very good and/or the model error is small, so the number of sensors needed is at most the number of sensors required for making the problem identifiable. For large measurement errors in Figure 5, the quality of information deteriorates significantly due to measurements and/or model error and so the minimum number of eight sensors required for identifiability appears less informative than the case of small measurement error. More than eight sensors increase further the utility values, providing significant additional information to counterbalance the deteriorated quality of the measurements.

Considering the cost of instrumentation, the process of placing more sensors optimally in the structure in order to gain a higher percentage of the total information should be considered with care and in some cases might not be justifiable (like in the case of small measurement and model error for the plate problem). Nevertheless, the final choice of the number of sensors to be placed in the structure depends on the cost of instrumentation which may also affect the location of sensors, especially for the cases where instrumentation cost depends on the location of a sensors. For example, not easily accessible areas in a structure, such as underwater locations in off-shore platforms or wind turbines, might substantially increase the cost of adding sensors in relation to the cost of instrumenting easily accessible areas. However, considering cost issues in designing the sensor configuration falls outside the objectives of this work and the reader is referred to value of information formulations (e.g., [72,73]).

**Figure 6:**
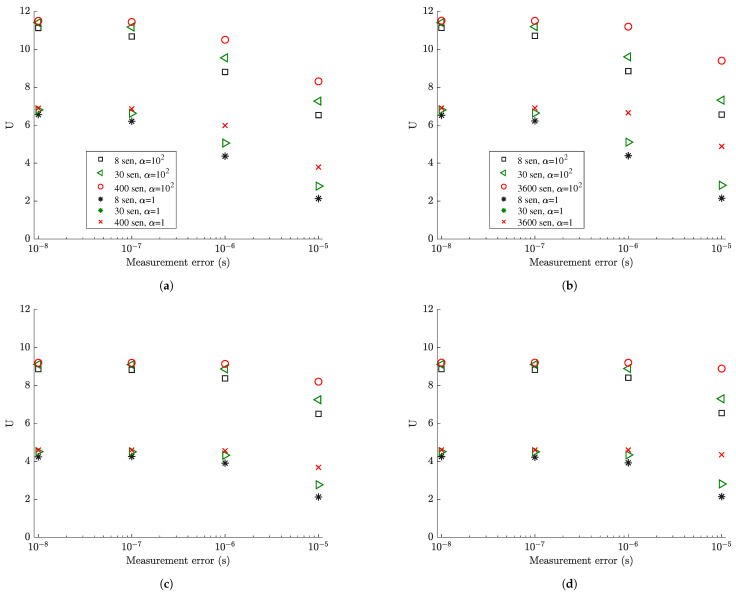
Information gain values versus measurement error for 8, 30 sensors and $N_{\mathrm{all}}$ sensors ($N_{\mathrm{all}}$ = 440 and 3660 respectively for the coarse and fine mesh), for $\alpha =10^{2}$ and α=1; (**a**,**b**) $\sigma_{e}=\sigma_{\varepsilon }=0.001$, (**c**,**d**) $\sigma_{e}=\sigma_{\varepsilon }=0.01$, (**a**,**c**) coarse mesh, (**b**,**d**) fine mesh.

**Figure 7:**
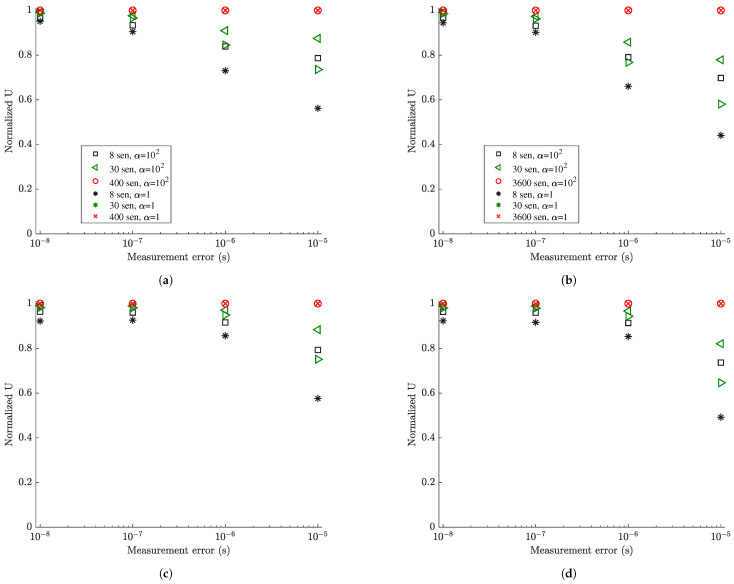
Normalized information entropy versus measurement error for 8, 30 and $N_{\mathrm{all}}$ sensors ($N_{\mathrm{all}}$ = 440 and 3660 respectively for the coarse and fine mesh), for $\alpha =10^{2}$ and α=1; (**a**,**b**) $\sigma_{e}=\sigma_{\varepsilon }=0.001$, (**c**,**d**) $\sigma_{e}=\sigma_{\varepsilon }=0.01$, (**a**,**c**) coarse mesh, (**b**,**d**) fine mesh.

Figure 6 and Figure 7 plots the information gain as a function of the measurement error *s* for 8, 30 and $N_{all}$ sensors, for both the coarse and the fine mesh, where $N_{all}$ is the number of finite elements in the coarse or fine mesh. For fixed number of sensors, the information gain decreases as the measurement error increases. This is due to the fact that the quality of information contained in measurements decreases due to higher noise to signal ratio and thus the information gain is lower as the measurement error increases. The decrease is more pronounced for very small modeling error ($\sigma_{e}=\sigma_{\epsilon }=0.001$) since most of the error in this case, modeled in the covariance matrix $Q_{e}$ in Equation (27), arises from the measurement error. For higher model error ($\sigma_{e}=\sigma_{\epsilon }=0.01$) shown in Figure 6c,d, the information gain values are less sensitive to the measurement error values of $s=10^{-9}$ (very small), $s=10^{-8}$ (small) and $s=10^{-7}$ (moderate), while there is a more pronounced drop in information gain for large measurement error ($s=10^{-6}$). This insensitivity of the information gain to smaller values of the measurement error is due to the fact that the larger value of model error dominates the very small to moderate measurement errors as seen by the mathematical model for $Q_{e}$ in Equation (30). The quality of information in the data will be further deteriorated only for sufficiently large values of measurement error (here $s=10^{-6}$).

Comparing the results in Figure 6 for values of α=1 and $\alpha =10^{2}$ corresponding to small and large prior uncertainty in the modal coordinates, it is clearly seen that the information gain for small prior uncertainty is less than the information gain for large prior uncertainty since significant part of the information is provided from the more informative (due to narrower bounds) prior distribution of the modal coordinates $\underline{\xi }\left(t\right)$, making the data effectively less informative. Comparing the results in Figure 7, the decrease in the percentage information gain, normalized with respect to the maximum information that can be achieved by fully populating the plate with strain sensors, is more pronounced as the measurement error increases. For example, 30 sensors placed at their optimal position using α=1 (narrower prior bounds) accounts for approximately 70% of the information that can be gained from strain sensor placement as opposed to approximately 90% of the percentage information gain that can be achieved with $\alpha =10^{2}$ (large prior uncertainty bounds). This is expected since in the case of α=1 the prior contains significant information in relation to the information provided from the data.

#### 6.1.5. Optimal Locations of Strain Sensors

**Figure 8:**
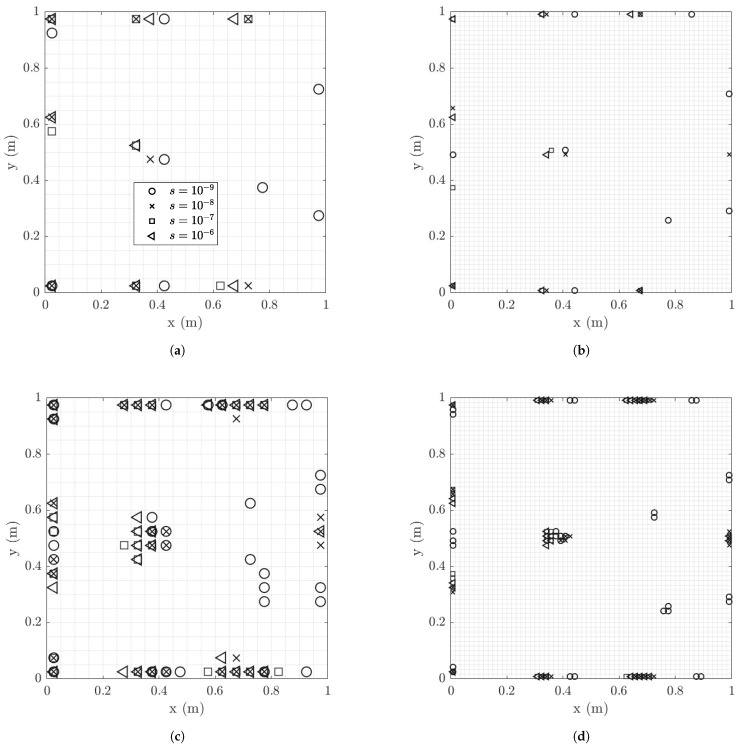
Best sensor positions obtained with model/prediction error $\sigma_{e}=\sigma_{\varepsilon }=0.001$ for 8 sensor for (**a**) coarse and (**b**) fine mesh, and for 30 sensor for (**c**) coarse and (**d**) fine mesh.

**Figure 9:**
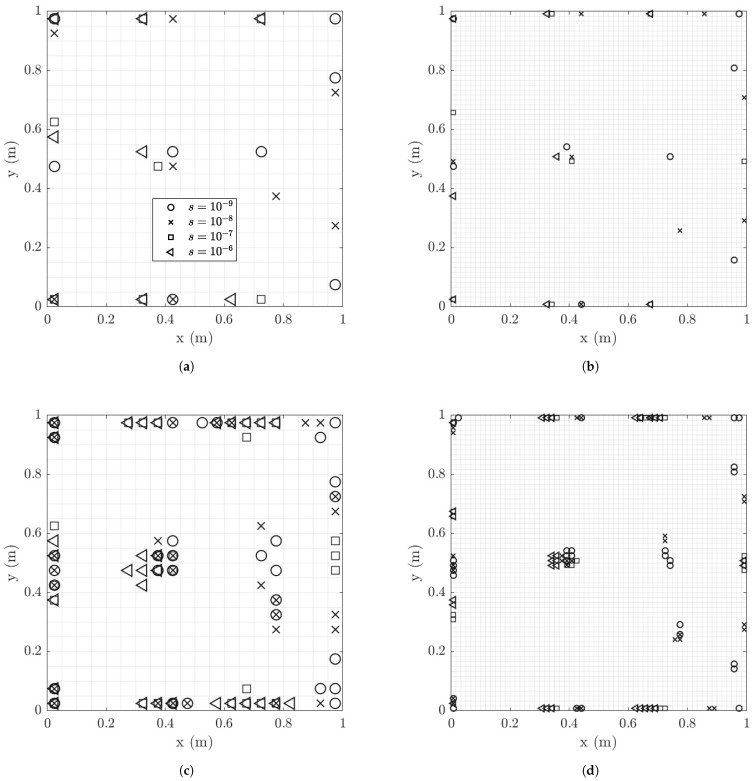
Best sensor positions obtained with model/prediction error $\sigma_{e}=\sigma_{\varepsilon }=0.01$ for 8 sensor for (**a**) coarse and (**b**) fine mesh, and for 30 sensor for (**c**) coarse and (**d**) fine mesh.

Optimal strain sensor positions for 8 and 30 sensors are shown in Figure 8 and Figure 9 for model/prediction errors $\sigma_{e}=\sigma_{\epsilon }=0.001$ and 0.01, respectively. The optimal sensor locations are compared for different values of the measurement errors. Comparing Figure 8a,b for eight sensors and Figure 8c,d for 30 sensors, it can be seen that the results for the coarse and the fine mesh are very similar for a given measurement error. For very small measurement error, sensors are placed towards the right edge of the plate where strains are small compared to the strains at the left side and middle area of the plate. The reason is that the OSP methodology for predicting strain responses in all finite elements of the plate has a tendency to spread the sensors to cover the whole surface of the plate as long as the quality of information is very good over the plate surface. In the very small measurement error case, the errors are much smaller than the intensity of the strains and so signal to noise ratio is high and most strain locations in the plate are informative. For large measurement errors, placing sensors in the right edge is avoided since the signal to noise ratio decreases and the quality of information from sensors placed towards the right edge is substantially deteriorated. For higher model/prediction error values of $\sigma_{e}=\sigma_{\epsilon }=0.01$ (see Figure 9) there is a tendency that the sensor move from left to the right, towards strains with smaller intensities. This is due to the fact that higher model/prediction error dominates the overall error, with the size of measurement error playing a lesser role in the optimal sensor placement design. In this case, since the model error is assigned in each position as a fraction of the intensity of the strains measured in the respective positions, all positions on the surface plate do provide similar information with the noise (here model error) to signal ratio to be the same, thus the sensors towards the left edge are equally counted in the optimal sensor placement methodology.

#### 6.1.6. Effect of Spatial Correlation of Model Error

It is observed in Figure 8c,d and Figure 9c,d that the 30 sensors placed optimally in the structure are clustered in specific regions on the plate surface. The size of each clustering region is proportional to the size of the finite element used for coarse and fine mesh. So clustering in similar for both coarse and fine mesh sizes. To avoid sensor clustering one has to use the spatial correlation function in Equation (29) [57] for the prediction error.

**Figure 10:**
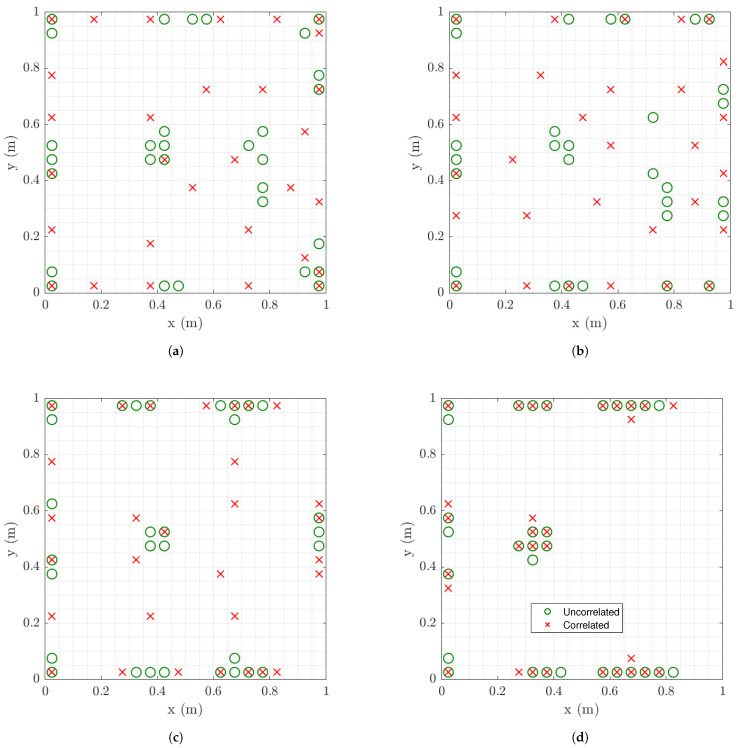
Comparison of optimal sensor placement for 30 sensors for the spatially uncorrelated (λ=0) and correlated (λ=0.1 cases; $\sigma_{e}=\sigma_{\varepsilon }=0.01$, $\alpha =10^{2}$ and measurement error (**a**) very small ($s=10^{-9}$), (**b**) small ($s=10^{-8}$), (**c**) moderate ($s=10^{-7}$), (**d**) large ($s=10^{-6}$).

The effect of correlation in the model error is next investigated as a function of the size of measurement and model error. Figure 10 compares the optimal sensor locations for 30 sensors for spatially uncorrelated (ρ=0) and correlated (ρ=0.1) prediction error models for different values of the measurement error and for model/prediction error equal to $\sigma_{e}=\sigma_{\varepsilon }=0.01$. The results clearly indicate that for the correlated case clustering is avoided and the 30 sensors are more uniformly distributed over the surface of the plate for relatively small to moderate measurement error. This is due to the fact that the model error dominates the prediction error for relatively small measurement error and thus the measurement error has a small effect on the design of optimal sensor locations. However, for large measurement error, clustering persists (Figure 10d) since the measurement error is the dominant source of error compared to the model error. Thus model error and as a result the effect of spatial correlation structure of the model error is insignificant and does not affect the design of the sensors for large ($s=10^{-6}$) values of the measurement error. So the clustering problem reappears and the model error correlation structure has no effect on the optimal sensor placement.

#### 6.1.7. Effectiveness of Optimal Sensor Configuration for Response Predictions

The effectiveness of the best sensor configuration is next investigated using simulated measurements. For this, simulated strain response time histories are generated from the model of the plate subjected to white noise input at location A (Figure 1) and using up to eight contributing modes. The simulations are generated using a sampling period Δt=0.01 s and the standard deviation $\sigma_{wn}=1$ of a Gaussian white noise sequence. To simulate measurement error (noise from sensors), zero mean Gaussian white noise with standard deviation 1% of the simulated response at each time instant is added to generate the noise contaminated measurements. Alternatively, to simulate model error, the measured data are simulated using a model with mass values for all finite elements randomly perturbed by adding to the nominal mass values zero-mean Gaussian distributed values with standard deviation equal to 5% of the nominal mass values.

The relative errors between the strain responses predicted by the modal expansion technique given a fixed number of sensors and the simulated measurements are used to demonstrate the effectiveness of the optimal sensor configuration in the presence of measurement or model error. The relative strain error at each location is defined as the ratio of the root mean square error ϵ between the predicted and measured responses over the root mean square value (intensity) of the measured strain response time history, given by

(38) $$\epsilon_{i,rel}=\frac{\sqrt{\frac{1}{N}\sum_{k=1}^{N}{\left[{\hat{z}}_{i}\left(k\right)-z_{i}\left(k\right)\right]}^{2}}}{\sqrt{\frac{1}{N}\sum_{k=1}^{N}{\left[z_{i}\left(k\right)\right]}^{2}}}$$

where *N* is the number of data points in the time histories, ${\hat{z}}_{i}\left(k\right)$ is the predicted values from the nominal model based on the modal expansion Equation (10), and $z_{i}\left(k\right)$ is the simulated “measurements” at the *i*-th DOF, and *k* indicates the time index corresponding to time instant $t_{k}=k\Delta t$.

**Figure 11:**
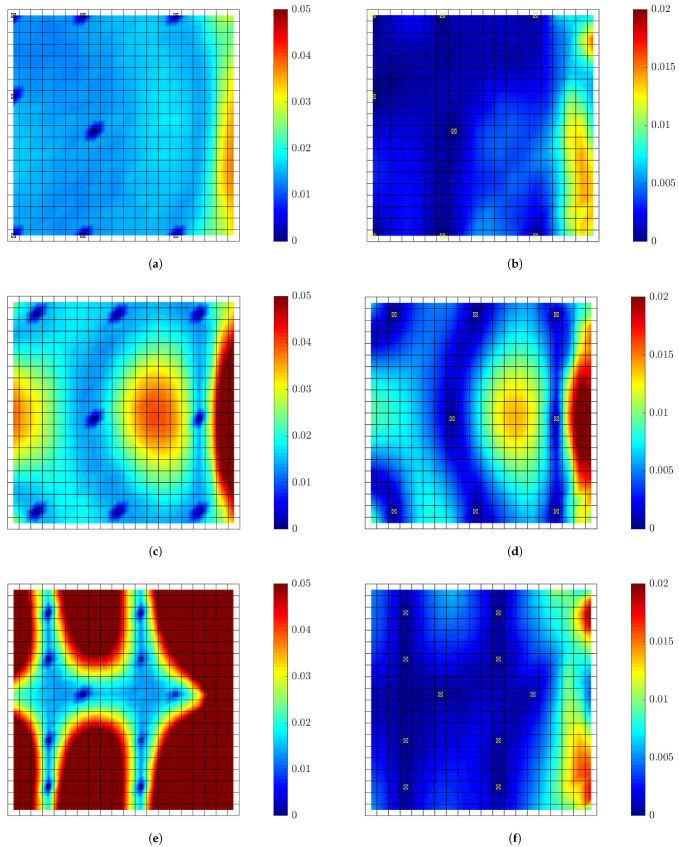
Relative error in response predictions with 8 optimally located strain sensors corresponding to information gain U= 8.38 (92%) (**a**,**b**), with alternative sensor locations with 8 sensors corresponding to information gain U= 7.4 (81%) (**c**,**d**) and with 10 sensors corresponding to information gain U= 7 (77%) (**e**,**f**); (**a**,**c**,**e**) for measurement error, (**b**,**d**,**f**) for model error. The location of sensors are shown with the box-cross symbols in each subfigure. ($\sigma_{e}=\sigma_{\varepsilon }=0.01$, $s=10^{-7}$ and $\alpha =10^{2}$).

Figure 11a,b presents the results for the relative errors of the optimal sensor configuration design for eight sensors corresponding to information gain value of U=8.38 (92% of the maximum that could be achieved by fully populating the plate surface with strain sensors), and for two alternative sub-optimal sensor configurations for eight sensors (Figure 11c,d)) corresponding to lower information gain value of U=7.4 (81%) and for higher number of 10 sensors (Figure 11e,f) also corresponding to lower information gain value of U=7 (80%). For the optimal sensor configuration cases the predictions are quite reliable with relative errors less than 2% and 0.8% over 90% of the surface of the plate for measurements simulated for noise and model error, respectively. The errors are higher over 10% of the surface close to the right edge where strain level are very low with high noise to signal ratio in the measured time histories. For both measurement errors (Figure 11a,c,e) and model/prediction error (Figure 11b,d,f), the predictions from the optimal sensor configuration (Figure 11a,b) are consistently better than the predictions obtained from the sub-optimal sensor configurations since the relative errors based on the optimal sensor configuration are overall lower than the relative errors obtained from the sub-optimal sensor configurations over the surface of the plate. In particular, for the case of measurement error, the predictions based on a sensor configuration with higher number of 10 sub-optimal sensors (Figure 11e) are significantly worse than the predictions from the optimal configuration containing less number of eight sensors (Figure 11a), emphasizing the need of a cost-effective design of the sensor network in a structure. It is also clear that the errors in response predictions obtained at the measured locations is lower than errors in the predictions at other non-measured locations. Finally, it should be noted that there exist sensor configurations corresponding to information gain values closer to the minimum information gain (not shown in the figures) that provide relative errors higher than 100% which means that predictions can be completely unreliable from such non-optimal sensor configurations.

**Figure 12:**
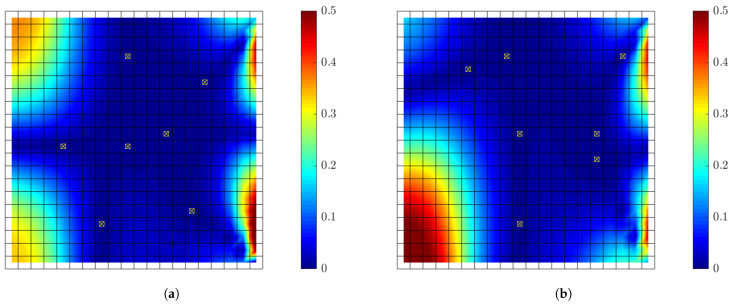
Relative error in response prediction with 7 best sensor positions (**a**) with $S=\alpha^{2}Q_{\xi }$ and (**b**) with $S=\alpha^{2}Q_{\xi }\left(1,1\right)I$. ($\sigma_{e}=\sigma_{\epsilon }=0.01$, $s=10^{-7}$ and $\alpha =10^{2}$).

Figure 12 presents relative error results for two optimal sensor configurations for seven sensors designed using large prior uncertainty ($\alpha =10^{2}$) in the modal coordinates and for two choices of the covariance matrix *S* of the prior distribution. The first case corresponds to covariance matrix of the prior proportional to the non-diagonal covariance matrix of the modal coordinates obtained for white noise input (see Equation (32)), while the second choice corresponds to diagonal isotropic covariance matrix with strength proportional to the variance of the first modal coordinate ($S=\alpha^{2}Q_{\xi }\left(1,1\right)I$). In the first case the relative intensity of the modal coordinates is taken into account in the definition of the prior covariance matrix, while in the second case this relative intensity is ignored and all modal coordinates are equally considered in the definition of the covariance matrix *S* of the prior distribution. It can be seen that the error distribution over the plate surface differs for the two optimal designs. Moreover, the relative errors in the predictions obtained from the first optimal sensor configuration are lower than the errors obtained from the second optimal sensor configuration, signifying the importance in considering the intensity of each mode, affected by the excitation frequency content, in the choice of the prior.

#### 6.1.8. Robustness to Model/Prediction and Measurement Error Uncertainties

Robust optimal sensor placement results are next obtained by taking into account the uncertainties in the model/prediction error parameters $\sigma_{e}$ and $\sigma_{\varepsilon }$ and the measurement error parameter *s*. Specifically, the re-parameterization $\sigma_{e}=\sigma_{\varepsilon }=10^{-\beta_{\sigma }}$ and $s=10^{-\beta_{s}}$ is used and uniform uncertainty is assigned in the values of $\beta_{\sigma }$ and $\beta_{s}$ with bounds that cover the previously lower and upper values of these parameters. The distributions are selected to be $\beta_{\sigma }∼U\left(2,3\right)$, $\beta_{s}∼U\left(6,9\right)$, where U(a,b) denotes a uniform distribution with lower bound *a* and upper bound *b*. This case accounts for the realistic scenario of uncertain model/prediction and measurement errors assigned in the formulation, arising mostly from the uncertain excitation intensities and frequency content that have to be taken into account in the design of the optimal sensor configuration. The uncertain parameters $\beta_{\sigma }$ and $\beta_{s}$ are included in the nuisance parameter set φ and their uncertainty is taken into account in the generalized utility function introduced in Equation (25). The sparse grid algorithm [86] of order 4 is used to evaluate the integrals in Equation (25).

**Figure 13:**
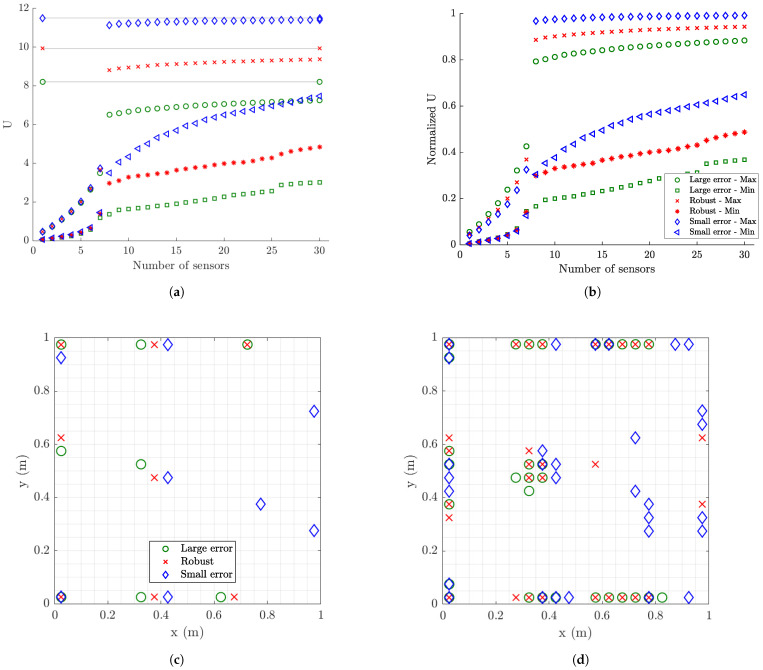
Comparison of best SSP robust results with best SSP results obtained from large and small model/prediction and measurement error cases for the coarse mesh and strain sensors; (**a**) maximum utility values, (**b**) normalized utility values; Optimal sensor placement for (**c**) 8 sensors, and (**d**) 30 sensors.

Results for the maximum robust information gain values are compared in Figure 13a,b with the corresponding maximum information gain values obtained by fixing the uncertain error parameters values ($\sigma_{e}$, $\sigma_{\varepsilon }$ and *s*) to the minimum values by selecting $\beta_{\sigma }=3$ and $\beta_{s}=9$, as well as maximum values by selecting $\beta_{\sigma }=2$ and $\beta_{s}=6$. The corresponding optimal sensor locations are compared in Figure 13c,d. It can be seen that the robust information gain estimates differ from the estimates obtained from the fixed minimum and maximum values of the model/prediction and measurement error parameters. As expected the results for the robust information gain values are found between the information gain values using the minimum or maximum values of the error parameters. The optimal sensor configuration proposed by the robust OSP methodology differs from the optimal sensor configuration obtained by the OSP methodology corresponding to minimum values of the error parameters. Specifically, for the very small model/prediction error case the sensors tend to be placed towards the right edge of the plate since, despite the smaller strain intensity in this area, the noise to signal ratio is very small and thus the measurements from this right edge are also informative. The robust OSP design seems to be closer to the OSP design corresponding to the maximum values of the error parameters. This is due to the high measurement and model/prediction errors that are taken into account in the assigned probability distribution of the error parameters. As a result, sensors designed according to the robust information gain are placed farther away from the right edge of the plate due to larger noise to signal ratio in the locations close to the right edge.

### 6.2. Strain Predictions Using Displacement Observations

**Figure 14:**
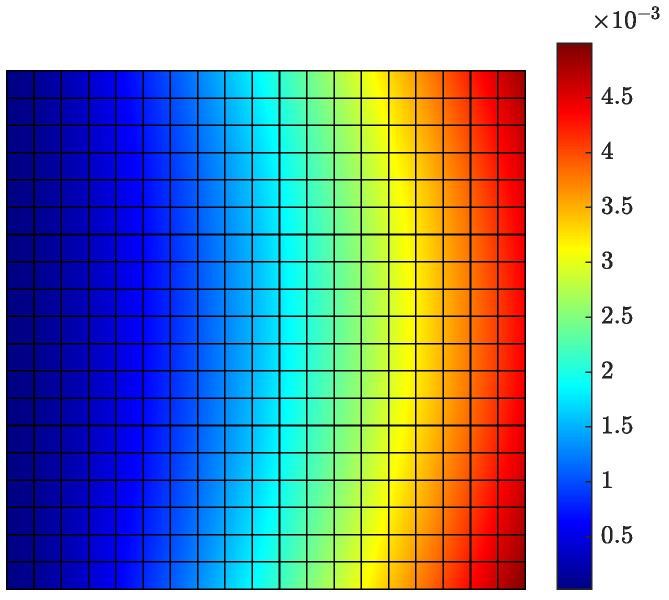
Intensities of the displacement observation $Q_{y}^{1/2}$ as a function of the number of modes for the coarse mesh.

The methodology is next applied to the case where displacement sensors are used for predicting strains at the midpoints of all finite elements of the coarse mesh. Displacement sensors measure out-of-plane displacements at the nodes of the mesh, perpendicular to the plate surface. As before, the number of contributing modes are kept to eight. For choosing the measurement and model/prediction error parameters, the intensities of the displacement responses at all nodes of the finite element mesh to a white noise excitation at the right lower corner of the plate (point A in Figure 1) are shown in Figure 14. Based on the results in this figure the measurement errors can now be selected as in Table 3.

Uncertainties in the model/prediction error parameters $\sigma_{e}$ and $\sigma_{\varepsilon }$ and the measurement error parameter *s* are accounted for in the sensor placement design. As before, the re-parameterization $\sigma_{e}=\sigma_{\varepsilon }=10^{-\beta_{\sigma }}$ and $s=10^{-\beta_{s}}$ is used and uniform uncertainty is assigned in the values of $\beta_{\sigma }$ and $\beta_{s}$ with bounds to cover the lower and upper values of these parameters shown in Table 3. Thus, the distributions are selected to be $\beta_{\sigma }∼U\left(2,3\right)$, $\beta_{s}∼U\left(4,7\right)$.

**Figure 15:**
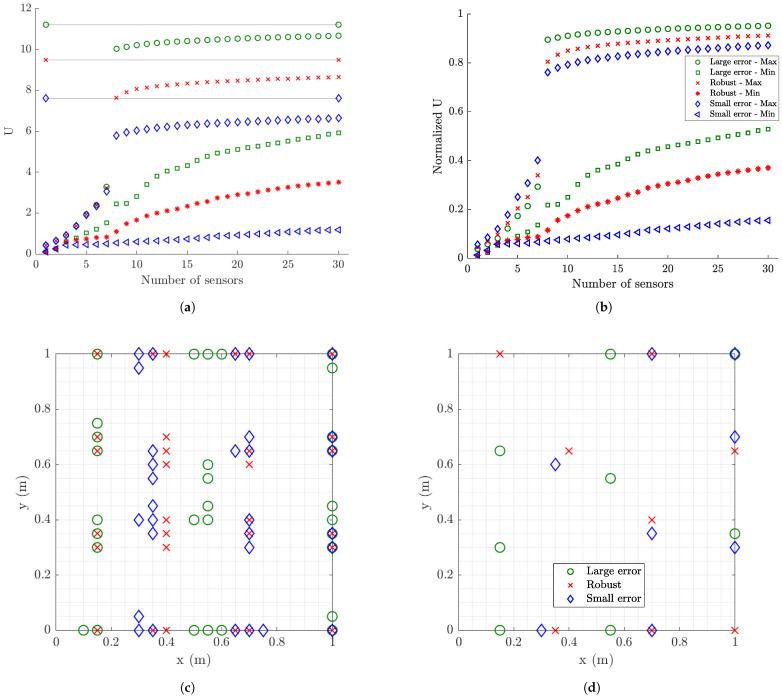
Comparison of best SSP robust results with best SSP results obtained from large and small model/prediction and measurement error cases for the coarse mesh and displacement sensors; (**a**) maximum utility values, (**b**) normalized utility values; Optimal sensor placement for (**c**) eight sensors, and (**d**) 30 sensors.

Results for the maximum robust information gain values are compared in Figure 15a,b with the corresponding maximum information gain values obtained for very small errors ($\beta_{\sigma }=3$ and $\beta_{s}=7$) as well as large errors ($\beta_{\sigma }=2$ and $\beta_{s}=4$). The corresponding optimal sensor locations are compared in Figure 15c for eight sensors and Figure 15d for 30 sensors. Comparing the results for the robust error case with the ones for small and large error cases, the results are found to be qualitatively similar to the ones obtained for strain sensor measurements in Figure 13. Specifically, the robust sensor placement design differs significantly from the design based on small error case, while it is closer to the sensor placement design based on the large error case. The optimal location of displacement sensors for the small error case tends to also cover the left fixed edge of the plate where displacements are relatively small compared to the middle and right locations of the plate since the noise to signal ratio from these displacement locations is small and thus the measured displacements, despite their relative small values, are informative. The optimal sensor placement for large error tends to move towards the left edge of the plate where the displacements are usually large and the noise to signal ratio is small. The locations close to the left edge (fixed support) of the plate are avoided in this case due to high noise to signal ratio for large measurement errors.

**Figure 16:**
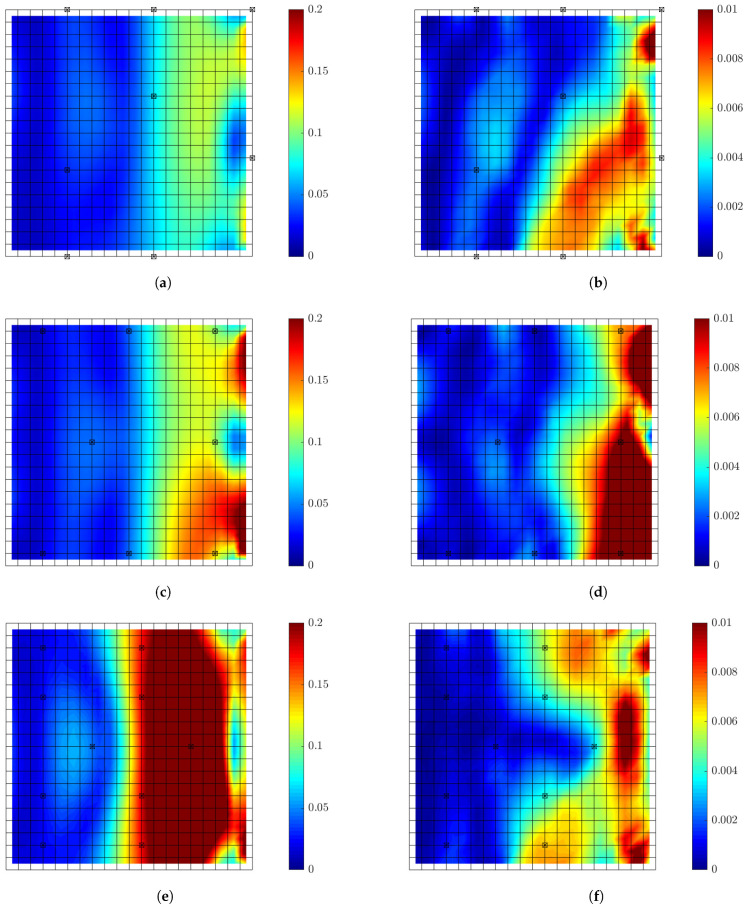
Relative error in response prediction with eight optimally located displacement sensors corresponding to information gain U=7.3 (84%) (**a**,**b**), with alternative sensor locations with eight sensors corresponding to information gain U=6.8 (78%) (**c**,**d**) and with 10 sensors corresponding to information gain U=6.6 (75%) (**e**,**f**); (**a**,**c**,**e**) for measurement error, (**b**,**d**,**f**) for model error. The location of sensors are shown with the box-cross symbols in each sub-figure. ($\sigma_{e}=\sigma_{\epsilon }=0.01$, $s=10^{-5}$).

Figure 16a,b presents the results for the relative errors for the optimal displacement sensor configuration for 8 sensors corresponding to information gain value of U=7.3 (84%), and for two alternative sub-optimal sensor configurations for 8 sensors (Figure 16c,d) corresponding to lower information gain value of U=6.8 (78%) and for higher number of 10 sensors (Figure 16e,f) also corresponding to lower information gain value of U=6.6 (75%). Comparing the effectiveness of the optimal and the two sub-optimal sensor configurations, the results are qualitatively similar to the ones presented in Figure 11a,b for the strain sensor case. The relative errors for the optimal sensor configurations are lower than the relative errors for the two sub-optimal sensor configurations for both 8 and 10 sensors and for both measurement (Figure 16a,c,e) and model (Figure 16b,d,f) errors, pointing out the superiority of the optimal sensor configuration for reliable response predictions.

Comparing Figure 15a,b with Figure 13a,b, it is clear that the information gain values for strain sensors in Figure 13a,b are higher than the information gain values for displacement sensors in Figure 15a,b. Thus, among the two type of strain and displacement sensors it is preferred to place in the structure strain sensors. This is also confirmed from the relative errors values for the optimal sensor configuration obtained for the displacement sensors in Figure 16a,b. These relative errors reach values as high as 13% and 1% for the simulated measurement error and the model/prediction error cases, respectively, which are higher than the corresponding values of 2% and 0.8% for strain sensors presented in Figure 11a,b. However, this result holds for the specific plate structure analysed and cannot be generalized for other applications. An investigation for the best combination of displacement and strain sensors has to be performed for each application. The methodology proposed in this work can be extended to fuse sensors by optimally placing simultaneously displacement and strain sensors for gaining the maximum information for response predictions with the smallest uncertainty.

## 7. Conclusions

Using information and utility theory, the optimal sensor placement problem for reliable virtual sensing and response reconstruction is formulated based on the modal expansion technique as a problem of maximizing a multi-dimensional integral quantifying the information gain from the data. The framework addresses the challenging case of output-only vibration measurements and provides optimal sensor configurations that are robust to uncertainties in model parameters as well as in model/prediction and measurement errors. Such uncertainties are usually not known in the initial optimal experimental design phase and thus need to be postulated using prior distributions. Sparse grid or Monte Carlo techniques can be used to estimate the multidimensional integral that arises in the robust formulation. Computationally efficient heuristic forward and backward sequential sensor placement strategies are combined to estimate the near optimal sensor locations. Useful expressions are derived for the effect of measurement and model/prediction errors on the information gained by a sensor configuration. As these errors increase, it was shown analytically that the information gain decreases. In addition, it was analytically derived that the information increases as one adds sensors in the structure, as it would be intuitively expected.

The methodology was demonstrated by designing the optimal strain or displacement sensors over a plate model of a structure. A thorough investigation was conducted on the effect of measurement and model/prediction errors, the size of the prior uncertainty in the modal coordinates, the spatial correlation structure of the model error, the uncertainties in model/prediction and measurement errors on the optimal sensor placement and the variation of the highest and lowest utility (information gain) values as a function of the number of sensors. The issue of the effect of the excitation characteristics on the design of the optimal sensor configuration was also pointed out. Excitation characteristics (locations, intensity and frequency content of excitations) affect response intensities and thus the selection of the level of measurement errors due to sensor accuracy and the model/prediction error levels due to different mechanisms activated/re-activated from different excitation levels and/or excitation frequencies. It was demonstrated that sensor accuracy (measurement error), related to noise to signal ratio, affected the optimal placement of sensors. The model/prediction error has also an effect on the optimal design. In particular, when model/prediction error dominates the measurement error, the accuracy of the sensors plays insignificant role in the design of the optimal sensor configuration. The level of noise to signal ratio are not known a priori since the intensity and frequency content of the excitation and thus the level of measured response is not known. The size of model/prediction errors due to model inadequacy is also not known a priori. Thus a robust design is more rational to use in order to better account for uncertainty in measurements and model/prediction errors. Such robust design over wide uncertainty bounds of errors leads to optimal sensor placement designs that are closer to the ones obtained for high measurement and model/prediction error, provided that measurement error dominates the model/prediction error. The effectiveness of the optimal designs was validated against sub-optimal ones by comparing errors in the predictions between the modal expansion method and simulated, noise and/or model error contaminated, measurements. It was also found that strain measurements are slightly more informative than displacement measurements for virtual strain sensing for the specific application. The proposed information-based method can be extended to select the optimal sensor configuration that contains both displacement and strain sensors.

The proposed OSP methodology is appropriate to use for reliably reconstructing responses that are important for providing data-driven safety and performance estimates of systems, as well as reconstructing stress response time histories that are needed for predicting fatigue damage accumulation.

## Figures and Tables

**Table 1:** Modal frequencies of the plate modeled with coarse and fine mesh.

Natural Frequency (Hz)	Mode 1	Mode 2	Mode 3	Mode 4	Mode 5	Mode 6	Mode 7	Mode 8
Coarse mesh	0.956	2.344	5.897	7.520	8.563	14.989	17.176	17.895
Fine mesh	0.956	2.344	5.868	7.497	8.532	14.934	16.909	17.696
% difference	0.00	0.00	0.49	0.31	0.36	0.37	1.55	1.11

**Table 2:** Different measurement errors *s* assumed. $\epsilon_{\min}=Q_{z,min}^{1/2}\approx 10^{-6}$ is the minimum value of the element strain that cover 98% of the plate surface. $\epsilon_{\max}=Q_{z,max}^{1/2}\approx 2\times 10^{-5}$ is the maximum value of the strain in the plate surface.

Measurement Error	*s*	$s/\varepsilon_{\min}$	$s/\varepsilon_{\max}$
Very small	$10^{-9}$	$10^{-3}$	$5\times 10^{-5}$
Small	$10^{-8}$	$10^{-2}$	$5\times 10^{-4}$
Moderate	$10^{-7}$	$10^{-1}$	$5\times 10^{-3}$
Large	$10^{-6}$	$10^{0}$	$5\times 10^{-2}$

**Table 3:** Different measurement errors assumed. $d_{\min}=Q_{z,min}^{1/2}\approx 10^{-4}$ is the minimum value of the node displacements to discrete white noise input with $\sigma_{wn}$ that cover 92% of the plate surface. $d_{\max}=Q_{z,max}^{1/2}\approx 5\times 10^{-3}$ is the maximum value of the displacement to same white noise input.

Measurement Error	*s*	$s/d_{\min}$	$s/d_{\max}$
Very small	$10^{-7}$	$10^{-3}$	$2\times 10^{-5}$
Small	$10^{-6}$	$10^{-2}$	$2\times 10^{-4}$
Moderate	$10^{-5}$	$10^{-3}$	$2\times 10^{-4}$
Large	$10^{-4}$	$10^{0}$	$2\times 10^{-2}$

## Abbreviations

The following abbreviations are used in this manuscript:

OSP	Optimal sensor placement
QoI	Quantities of interest
KL-div	Kullback-Leibler divergence
DOF	Degrees of freedom
FSSP	Forward sequential sensor placement
BSSP	Backward sequential sensor placement
SSP	Sequential sensor placement
CMA-ES	Covariance matrix adaptation evolution strategy
FIM	Fisher information matrix

## Nomenclature

$\underline{\varphi }$	structural model parameters
$n_{z}$	number of response locations
$\underline{u}$	excitation vector
$n_{u}$	number of excitation
*D*	the available data
$\underline{y}$	vector of response time history data
$N_{0}$	number of sensors
$\underline{\delta }$	sensor configuration vector
$\underline{z}$	vector of predicted responses
$\underline{\xi }$	vector of modal coordinates
*m*	number of modal coordinates
*n*	number of model degree of freedoms
*L*	observation matrix that maps the displacements at all *n* model DOF to the measured QoI indicated by the sensor location vector $\underline{\delta }$
Φ	displacement mode shape matrix
$\underline{e}$	a multi-variable zero-mean Gaussian noise term for measurement and model errors
$Q_{e}$	covariance matrix of $\underline{e}$
$\omega_{r}$	*r*-th modal frequency
Λ	diagonal matrix of the squares of the modal frequencies $\omega_{r}$
$\zeta_{r}$	*r*-th modal damping ratio
*Z*	diagonal matrix with diagonal elements equal to $2\omega_{r}\zeta_{r}$, r=1,…,m
*M*	a matrix of zeros and ones associating the independent excitations in the vector $\underline{u}\left(t\right)$ to the DOF of the structural model
Ψ	mode shape matrix of predicted QoI
$\underline{\varepsilon }$	a zero-mean prediction error term for model error
$Q_{\varepsilon }$	covariance matrix of $\underline{\varepsilon }$
$p(\underline{\xi }|\underline{y},\underline{\delta },\underline{\varphi })$	posterior PDF of the modal vector parameter $\underline{\xi }$ given $\underline{y}$, $\underline{\delta }$ and $\underline{\varphi }$
$p(\underline{\xi }|\underline{\varphi })$	prior PDF of $\underline{\xi }$ given $\underline{\varphi }$
$p(\underline{y}|\underline{\xi },\underline{\delta },\underline{\varphi })$	likelihood function of observing the data $\underline{y}$
$\Sigma_{\xi |D}$	posterior covariance matrix of the $\underline{\xi }$
$\Sigma_{z|D}$	posterior covariance matrix of the $\underline{z}$
$\Sigma_{\xi }$	covariance matrix of the assigned prior distribution for ξ
$w_{i}$	weight that quantifies the importance of the *i*-th QoI $z_{i}$
*U*	expected utility function
$H_{z}$	prior information entropy
$H_{z|D}$	posterior information entropy
$r(\underline{\delta },\underline{\varphi })$	ratio between $\Sigma_{z_{i}|D}$ and $\Sigma_{z_{i}}$
$\Delta {\bar{H}}_{z|D}\left(\underline{\delta }\right)$	Difference between prior and posterior information entropy for a sensor configuration $\underline{\delta }$
$N_{all}$	number of all possible sensor positions
$N_{F}$	number of function evaluations required by FSSP
$N_{B}$	number of function evaluations required by BSSP
${\underline{e}}_{meas}$	measurement error term in $\underline{e}$
${\underline{e}}_{model}$	model error term in $\underline{e}$
$Q_{e,meas}$	covariance matrix of ${\underline{e}}_{meas}$
$Q_{e,model}$	covariance matrix of ${\underline{e}}_{model}$
$\sigma_{\varepsilon }$	level of model error in $Q_{\varepsilon }$
$\sigma_{e}$	level of model error in $Q_{e,model}$
*s*	level of model error in $Q_{e,meas}$
*R*	the correlation matrix
α	a constant that quantifies the extent of the uncertainty in the prior distribution
*A*,*B*,*C*,*D*	state-space matrices
$Q_{x}$	covariance of the state vector
$\sigma_{wn}^{2}$	variance of the discrete white noise excitation
$\beta_{\sigma }$	parameter quantifying the uncertainty in $\sigma_{e}$ and $\sigma_{\varepsilon }$
$\beta_{s}$	parameter quantifying the uncertainty in *s*

## Appendix A. Proof of Equation (18)

Substituting Equation (14) into the inner integral of Equation (17) and using the properties of the logarithm (ln(A/B)=lnA−lnB), one has

(A1) $$\begin{array}{lll} \int_{Y}{\tilde{D}}_{i}\left(\underline{\delta },\underline{\varphi },\underline{y}\right)p\left(\underline{y},\underline{\varphi }|\underline{\delta }\right)d\underline{y} & = & \int_{Y}\int_{Z}p\left(z_{i}|\underline{y},\underline{\delta },\underline{\varphi }\right)\ln p\left(z_{i}|\underline{y},\underline{\delta },\underline{\varphi }\right)d{\underline{z}}_{i}p\left(\underline{y}|\underline{\varphi },\underline{\delta }\right)d\underline{y}\\ & & -\int_{Z}\left[\int_{Y}p\left(z_{i}|\underline{y},\underline{\delta },\underline{\varphi }\right)p\left(\underline{y}|\underline{\varphi },\underline{\delta }\right)d\underline{y}\right]\ln p\left(z_{i}|\underline{\varphi }\right)d{\underline{z}}_{i}\\ & = & \int_{Y}\int_{Z}p\left(z_{i}|\underline{y},\underline{\delta },\underline{\varphi }\right)\ln p\left(z_{i}|\underline{y},\underline{\delta },\underline{\varphi }\right)d{\underline{z}}_{i}p\left(\underline{y}|\underline{\varphi },\underline{\delta }\right)d\underline{y} \end{array}$$

(A2) $$\begin{array}{ccc} & & -\int_{Z}p\left(z_{i}|\underline{\varphi },\underline{\delta }\right)\ln p\left(z_{i}|\underline{\varphi }\right)d{\underline{z}}_{i} \end{array}$$

where the last equality is obtained by interchanging the order of integration of the double integral in the second term of Equation (A1) and noting that

(A3) $$\int_{Y}p\left(z_{i}|\underline{y},\underline{\delta },\underline{\varphi }\right)p\left(\underline{y}|\underline{\varphi },\underline{\delta }\right)d\underline{y}=p\left(z_{i}|\underline{\delta },\underline{\varphi }\right)$$

Introducing the prior information entropy

(A4) $$H_{z_{i}}\left(\underline{\varphi }\right)=-\int_{Z}p\left(z_{i}|\underline{\varphi },\underline{\delta }\right)\ln p\left(z_{i}|\underline{\varphi }\right)d{\underline{z}}_{i}$$

of $z_{i}\left(t\right)$ given the model parameter set $\underline{\varphi }$, and the posterior information entropy

(A5) $$H_{z_{i}|D}\left(\underline{y},\underline{\delta },\underline{\varphi }\right)=-\int_{Z}p\left(z_{i}|\underline{y},\underline{\delta },\underline{\varphi }\right)\ln p\left(z_{i}|\underline{y},\underline{\delta },\underline{\varphi }\right)d{\underline{z}}_{i}$$

of $z_{i}\left(t\right)$ given the data $\underline{y}$ and the model parameter set $\underline{\varphi }$, the integral in Equation (A2) simplifies to

(A6) $$\begin{array}{c} \int_{Y}{\tilde{D}}_{i}\left(\underline{\delta },\underline{\varphi },\underline{y}\right)p\left(\underline{y},\underline{\varphi }|\underline{\delta }\right)d\underline{y}=-\int_{Y}H_{z_{i}|D}\left(\underline{y},\underline{\delta },\underline{\varphi }\right)p\left(\underline{y}|\underline{\varphi },\underline{\delta }\right)d\underline{y}+H_{z_{i}}\left(\underline{\varphi }\right) \end{array}$$

Equation (18) is readily obtained by substituting Equation (A6) into Equation (17).

## Appendix B. Properties of Information Gain (Utility Function)

For notational convenience, in the following analysis the dependence of the quantities on the uncertain parameter set $\underline{\varphi }$ is dropped. From the mathematical structure of the covariance matrix $\Sigma_{z_{i}|D}\left(\underline{\delta }\right)$ appearing in Equation (12), one can readily observe that the quantity $\Sigma_{z_{i}|D}\left(\underline{\delta }\right)$ is non-negative. Additionally, for four matrices $A_{1}\in R^{n\times n}$, $B_{1}\in R^{m\times m}$, $V\in R^{m\times n}$ and $U\in R^{m\times n}$, the following useful property for the inverse of the sum of two matrices holds

(A7) $${\left(U^{T}B_{1}V+A_{1}\right)}^{-1}=A_{1}^{-1}-A_{1}^{-1}U^{T}{\left(B_{1}+VA_{1}^{-1}U^{T}\right)}^{-1}VA_{1}^{-1}$$

Setting $A_{1}=S^{-1}$, $B_{1}=Q_{e}^{-1}$, $U=V=L(\underline{\delta })\Phi$ and applying Equation (A7), the covariance matrix $\Sigma_{z_{i}|D}\left(\underline{\delta }\right)$ in Equation (12) can be written in the alternative form

(A8) $$\Sigma_{z_{i}|D}\left(\underline{\delta }\right)={\underline{\psi }}_{i}^{T}S{\underline{\psi }}_{i}-{\underline{\psi }}_{i}^{T}S\Phi^{T}L^{T}\left(\underline{\delta }\right){\left[L\left(\underline{\delta }\right)\Phi S\Phi^{T}L^{T}\left(\underline{\delta }\right)+Q_{e}\right]}^{-1}L\left(\underline{\delta }\right)\Phi S{\underline{\psi }}_{i}+Q_{\varepsilon_{i}}$$

The ratio between the posterior and prior covariance matrices in Equation (24) can thus be simplified in the form

(A9) $$r_{z_{i}|D}\left(\underline{\delta }\right)=1-\frac{{\left(L\left(\underline{\delta }\right)\Phi S{\underline{\psi }}_{i}\right)}^{T}{\left[L\left(\underline{\delta }\right)\Phi S\Phi^{T}L^{T}\left(\underline{\delta }\right)+Q_{e}\right]}^{-1}\left(L\left(\underline{\delta }\right)\Phi S{\underline{\psi }}_{i}\right)}{{\underline{\psi }}_{i}^{T}S{\underline{\psi }}_{i}+Q_{\varepsilon_{i}}}$$

Taking into account that the matrices *S* and $L\left(\underline{\delta }\right)\Phi S\Phi^{T}L^{T}\left(\underline{\delta }\right)+Q_{e}$ are semi-positive definite and that $Q_{\varepsilon_{i}}>0$, it can be concluded that the second term in Equation (A9) is non-negative and thus the value of the ratio is always less than or equal to one, i.e., $r_{z_{i}|D}\left(\underline{\delta }\right)\leq 1$. As a result, the utility function, quantifying the information gain from the data for least uncertainty in the response prediction, is always greater than or equal to zero.

### Appendix B.1. Effect of Modelling and Measurement Errors

Next we examine the effects of measurement and models errors quantified by the matrices $Q_{e}$ and $Q_{\varepsilon }$. The higher the value of the prediction error variance $Q_{\varepsilon_{i}}$, the higher the value of the denominator ${\underline{\psi }}_{i}^{T}S{\underline{\psi }}_{i}+Q_{\varepsilon_{i}}$ in Equation (A9) and the smaller the information gained by the sensor configuration. Let now $Q_{e}^{{}^{\prime }}$ be an error covariance matrix similar to Equation (30), corresponding to higher values of the measurement and model error, so that it admits the representation $Q_{e}^{{}^{\prime }}=Q_{e}+\Delta Q_{e}$, where $\Delta Q_{e}$ is positive definite matrix. This representation is true for the case where the values of $\sigma_{e}$ and/or $\sigma_{em}$ in Equation (30) are increased to represent higher measurement and/or modeling error. Substituting $Q_{e}^{{}^{\prime }}$ in place of $Q_{e}$ in Equation (A9) and using the expansion Equation (A7) for the inverse of $\left[L\left(\underline{\delta }\right)\Phi S\Phi^{T}L^{T}\left(\underline{\delta }\right)+Q_{e}^{{}^{\prime }}\right]=\left[L\left(\underline{\delta }\right)\Phi S\Phi^{T}L^{T}\left(\underline{\delta }\right)+Q_{e}+\Delta Q_{e}\right]$ by setting $A=[L\left(\underline{\delta }\right)\Phi S\Phi^{T}L^{T}\left(\underline{\delta }\right)+Q_{e}]$, $B=\Delta Q_{e}$ and U=V=I, the following expression for the new ratio $r_{z_{i}|D}^{{}^{\prime }}\left(\underline{\delta }\right)$ corresponding to the error covariance matrix $Q_{e}^{{}^{\prime }}$ arises

(A10) $$r_{z_{i}|D}^{{}^{\prime }}\left(\underline{\delta }\right)=r_{z_{i}|D}\left(\underline{\delta }\right)+\frac{{\underline{g}}^{T}{\left[\Delta Q_{e}+{\left(L\left(\underline{\delta }\right)\Phi S\Phi^{T}L^{T}\left(\underline{\delta }\right)+Q_{e}\right)}^{-1}\right]}^{-1}\underline{g}}{{\underline{\psi }}_{i}^{T}S{\underline{\psi }}_{i}+Q_{\varepsilon_{i}}}$$

where $\underline{g}={\left(L\left(\underline{\delta }\right)\Phi S\Phi^{T}L^{T}\left(\underline{\delta }\right)+Q_{e}\right)}^{-1}\left(L\left(\underline{\delta }\right)\Phi S{\underline{\psi }}_{i}\right)$. The numerator in the second term in the right hand side of Equation (A10) is positive due to the fact that both the matrices $\Delta Q_{e}$ and $\left(L\left(\underline{\delta }\right)\Phi S\Phi^{T}L^{T}\left(\underline{\delta }\right)+Q_{e}\right)$ are positive definite matrices. Thus the ratio $r_{z_{i}|D}^{{}^{\prime }}\left(\underline{\delta }\right)>r_{z_{i}|D}\left(\underline{\delta }\right)$ which implies that the information gain decreases as the measurement and model/prediction errors increases. As the model or measurement errors $\sigma_{e}$, $\sigma_{\varepsilon }$ and $\sigma_{em}$ become very large (in the limit as errors approach infinity), the ratio $r_{z_{i}|D}\left(\underline{\delta }\right)$ between the posterior and prior covariance matrices approach zero and the information gain is zero, independent of the number of sensors placed in the structure.

### Appendix B.2. Utility Versus Number of Sensors

Consider a sensor configuration $\underline{\delta }$ and a new sensor configuration ${\underline{\delta }}_{1}$ which consists of the sensors in the configuration $\underline{\delta }$ and one additional sensor placed in the structure. We will show that the utility value for the sensor configuration ${\underline{\delta }}_{1}$, containing an extra sensor, cannot be lower than the utility value of the sensor configuration $\underline{\delta }$, that is, $U_{i}\left({\underline{\delta }}_{1}\right)\geq U_{i}\left(\underline{\delta }\right)$. Let $L({\underline{\delta }}_{1})$ and ${\tilde{Q}}_{e}$ be the sensor locator matrix and the covariance error matrix in Equation (24) for $r_{z_{i}|D}\left({\underline{\delta }}_{1}\right)$ corresponding to the augmented sensor configuration ${\underline{\delta }}_{1}$. Then one has the partitions

(A11) $$L^{T}\left({\underline{\delta }}_{1}\right)=\left[\begin{array}{cc} L^{T}\left(\underline{\delta }\right) & {\underline{\ell }}_{1} \end{array}\right],{\tilde{Q}}_{1,e}=\left[\begin{array}{cc} Q_{e} & {\underline{q}}_{1}\\ {\underline{q}}_{1}^{T} & Q_{0} \end{array}\right]\mathrm{and}{\tilde{Q}}_{1,e}^{-1}=\left[\begin{array}{cc} {\tilde{Q}}_{e}^{-1} & {\underline{p}}_{1}\\ {\underline{p}}_{1}^{T} & p_{0}^{-1} \end{array}\right]$$

where using the properties of the inverse of a partitioned matrix ${\tilde{Q}}_{e}$ one has

(A12) $$\begin{array}{ccc} p_{0} & = & Q_{0}-{\underline{q}}_{1}^{T}Q_{e}^{-1}{\underline{q}}_{1} \end{array}$$

(A13) $$\begin{array}{ccc} {\tilde{Q}}_{e} & = & Q_{e}-{\underline{q}}_{1}Q_{0}^{-1}{\underline{q}}_{1}^{T} \end{array}$$

(A14) $$\begin{array}{ccc} {\tilde{Q}}_{e}^{-1} & = & Q_{e}^{-1}+\Delta {\tilde{Q}}_{e} \end{array}$$

(A15) $$\begin{array}{ccc} \Delta {\tilde{Q}}_{e} & = & Q_{e}^{-1}{\underline{q}}_{1}{\left[Q_{0}-{\underline{q}}_{1}^{T}Q_{e}^{-1}{\underline{q}}_{1}\right]}^{-1}{\underline{q}}_{1}^{T}Q_{e}^{-1} \end{array}$$

and ${\underline{p}}_{1}$ depends on the partitions of ${\tilde{Q}}_{1,e}$. Equation (A13) was derived using the identity in Equation (A7). Using the third of Equation (A11) and Equation (A13), the quantity $L^{T}\left({\underline{\delta }}_{1}\right)Q_{1,e}^{-1}L\left({\underline{\delta }}_{1}\right)$ involved in the ratio $r_{z_{i}|D}\left({\underline{\delta }}_{1}\right)$ simplifies to

(A16) $$\begin{array}{lll} L^{T}\left({\underline{\delta }}_{1}\right)Q_{1,e}^{-1}L\left({\underline{\delta }}_{1}\right) & = & \left[\begin{array}{cc} L^{T}\left(\underline{\delta }\right) & {\underline{\ell }}_{1} \end{array}\right]\left[\begin{array}{cc} {\tilde{Q}}_{e}^{-1} & {\underline{p}}_{1}\\ {\underline{p}}_{1}^{T} & p_{0}^{-1} \end{array}\right]\left[\begin{array}{c} L({\underline{\delta }}_{1})\\ {\underline{\ell }}_{1}^{T} \end{array}\right] \end{array}$$

(A17) $$\begin{array}{ccc} & = & \left[\begin{array}{cc} L^{T}\left(\underline{\delta }\right) & {\underline{\ell }}_{1} \end{array}\right]\left[\begin{array}{cc} Q_{e}^{-1} & \underline{0}\\ {\underline{0}}^{T} & 0 \end{array}\right]\left[\begin{array}{c} L(\underline{\delta })\\ {\underline{\ell }}_{1}^{T} \end{array}\right]+L^{T}\left({\underline{\delta }}_{1}\right)\left[\begin{array}{cc} \Delta {\tilde{Q}}_{e}^{-1} & {\underline{p}}_{1}\\ {\underline{p}}_{1}^{T} & p_{0}^{-1} \end{array}\right]L\left({\underline{\delta }}_{1}\right) \end{array}$$

(A18) $$\begin{array}{ccc} & = & L^{T}\left(\underline{\delta }\right)Q_{e}^{-1}L\left(\underline{\delta }\right)+L^{T}\left({\underline{\delta }}_{1}\right)\Delta {\tilde{Q}}_{1,e}L\left({\underline{\delta }}_{1}\right) \end{array}$$

where

(A19) $$\Delta {\tilde{Q}}_{1,e}=\left[\begin{array}{cc} \Delta {\tilde{Q}}_{e}^{-1} & {\underline{p}}_{1}\\ {\underline{p}}_{1}^{T} & p_{0}^{-1} \end{array}\right]$$

Note that $p_{0}$ in Equation (A12) is positive since it is the diagonal element of the inverse of a covariance matrix. Thus $\Delta {\tilde{Q}}_{e}$ in Equation (A15) is a positive definite matrix and as a result $\Delta {\tilde{Q}}_{1,e}$ in Equation (A19) is also a positive definite matrix.

Substituting in the numerator in Equation (24) for the sensor configuration ${\underline{\delta }}_{1}$, setting $E=\Phi^{T}L^{T}\left(\underline{\delta }\right)Q_{e}^{-1}L\left(\underline{\delta }\right)\Phi +S^{-1}$ and using Equation (A7) with A=E, $B=\Delta {\tilde{Q}}_{1,e}$ and $U=V=L({\underline{\delta }}_{1})\Phi$, the ratio $r({\underline{\delta }}_{1})$ for the sensor configuration ${\underline{\delta }}_{1}$ takes the form

(A20) $$\begin{array}{ccc} r_{z_{i}|D}\left({\underline{\delta }}_{1}\right) & = & \frac{{\underline{\psi }}_{i}^{T}{\left[\Phi^{T}L^{T}\left(\underline{\delta }\right)Q_{e}^{-1}L\left(\underline{\delta }\right)\Phi +\Phi^{T}L^{T}\left({\underline{\delta }}_{1}\right)\Delta {\tilde{Q}}_{1,e}L\left({\underline{\delta }}_{1}\right)\Phi +S^{-1}\right]}^{-1}{\underline{\psi }}_{i}+Q_{\varepsilon_{i}}}{{\underline{\psi }}_{i}^{T}Q_{e}^{-1}{\underline{\psi }}_{i}+Q_{\varepsilon_{i}}}\\ & = & \frac{{\underline{\psi }}_{i}^{T}{\left[E+\Phi^{T}L^{T}\left({\underline{\delta }}_{1}\right)\Delta {\tilde{Q}}_{1,e}L\left({\underline{\delta }}_{1}\right)\Phi \right]}^{-1}{\underline{\psi }}_{i}+Q_{\varepsilon_{i}}}{{\underline{\psi }}_{i}^{T}Q_{e}^{-1}{\underline{\psi }}_{i}+Q_{\varepsilon_{i}}}\\ & = & \frac{{\underline{\psi }}_{i}^{T}E^{-1}{\underline{\psi }}_{i}-{\underline{\psi }}_{i}^{T}Z\left({\underline{\delta }}_{1}\right){\underline{\psi }}_{i}+Q_{\varepsilon }}{{\underline{\psi }}_{i}^{T}Q_{e}^{-1}{\underline{\psi }}_{i}+Q_{\varepsilon_{i}}}=\frac{{\underline{\psi }}_{i}^{T}E^{-1}{\underline{\psi }}_{i}+Q_{\varepsilon_{i}}}{{\underline{\psi }}_{i}^{T}Q_{e}^{-1}{\underline{\psi }}_{i}+Q_{\varepsilon_{i}}}-\frac{{\underline{\psi }}_{i}^{T}Z\left({\underline{\delta }}_{1}\right){\underline{\psi }}_{i}}{{\underline{\psi }}_{i}^{T}Q_{e}^{-1}{\underline{\psi }}_{i}+Q_{\varepsilon_{i}}}\\ & = & r_{z_{i}|D}\left(\underline{\delta }\right)U_{i}\left(\underline{\delta }\right)-\frac{{\underline{\psi }}_{i}^{T}Z\left({\underline{\delta }}_{1}\right){\underline{\psi }}_{i}}{{\underline{\psi }}_{i}^{T}Q_{e}^{-1}{\underline{\psi }}_{i}+Q_{\varepsilon_{i}}} \end{array}$$

where

(A21) $$Z\left({\underline{\delta }}_{1}\right)=E^{-1}\Phi^{T}L^{T}\left({\underline{\delta }}_{1}\right){\left[\Delta {\tilde{Q}}_{1,e}+L\left({\underline{\delta }}_{1}\right)\Phi E^{-1}\Phi^{T}L^{T}\left({\underline{\delta }}_{1}\right)\right]}^{-1}L\left({\underline{\delta }}_{1}\right)\Phi E^{-1}$$

from which it follows that $r_{z_{i}|D}\left({\underline{\delta }}_{1}\right)\leq r_{z_{i}|D}\left(\underline{\delta }\right)$ since the matrices $\Delta {\tilde{Q}}_{1,e}$, $Q_{e}$, *E* and thus $Z({\underline{\delta }}_{1})$ are symmetric and semi-positive definite. As a result $U_{i}\left({\underline{\delta }}_{1}\right)\geq U_{i}\left(\underline{\delta }\right)$ and thus the information gained by adding a sensor in an existing sensor configuration, which is consistent with intuition. Moreover, it is straightforward to conclude that the maximum information gain is an increasing function of the number of sensors.
